# Effects of Teriparatide in Patients with Osteoporosis in Clinical Practice: 42-Month Results During and After Discontinuation of Treatment from the European Extended Forsteo® Observational Study (ExFOS)

**DOI:** 10.1007/s00223-018-0437-x

**Published:** 2018-06-16

**Authors:** Nicola Napoli, Bente. L. Langdahl, Östen Ljunggren, Eric Lespessailles, George Kapetanos, Tomaz Kocjan, Tatjana Nikolic, Pia Eiken, Helmut Petto, Thomas Moll, Erik Lindh, Fernando Marin

**Affiliations:** 1 Division of Endocrinology and Diabetes, University Campus Bio-Medico, Alvaro del Portillo 21, 00128 Rome, Italy; 2 Aarhus University Hospital, Aarhus, Denmark; 3 Department of Medical Sciences, Uppsala University, Uppsala, Sweden; 4 University Orléans, Orléans, France; 5 Regional Hospital of Orléans, Orléans, France; 6 Papageorgiou General Hospital, Thessaloniki, Greece; 7 University Medical Centre, Ljubljana, Slovenia; 8 University Hospital, Zagreb, Croatia; 9 Department of Cardiology, Nephrology and Endocrinology, Hillerød Hospital, Hillerød, Denmark; 10 Faculty of Health and Medical Sciences, University of Copenhagen, Copenhagen, Denmark; 11 Eli Lilly and Company, Windlesham, UK

**Keywords:** Back pain, Fracture, Observational study, Osteoporosis, Quality of life, Teriparatide

## Abstract

This study aimed to describe clinical outcomes in patients prescribed teriparatide and followed up for 18 months after stopping the drug in real-life conditions. The Extended Forsteo® Observational Study analysed incident clinical fractures in 6-month intervals using logistic regression with repeated measures. Changes in back pain (visual analogue scale) and health-related quality of life (HRQoL; EQ-5D questionnaire) were analysed using mixed models for repeated measures. Patients were analysed if they had a post-baseline visit, regardless of whether and for how long they took teriparatide. Of 1531 patients analysed (90.7% female, mean age: 70.3 years), 76 (5.0%) never took teriparatide. Median treatment duration was 23.6 months. The adjusted odds of clinical fracture decreased by 47% in the > 12- to 18-month treatment period (*p* = 0.013) compared with the first 6-month period, with no statistically significant reduction in the > 18- to 24-month interval. The clinical fracture rate remained stable during the 18 months’ post-teriparatide, when approximately 98% of patients took osteoporosis medication (51% bisphosphonates). Clinical vertebral fractures were reduced at every time period compared with the first 6 months. Adjusted mean back pain scores decreased and EQ-5D scores increased significantly at each post-baseline observation. In a real-life clinical setting, the risk of clinical fractures declined during 24 months of teriparatide treatment. This reduction was maintained 18 months after stopping teriparatide. In parallel, patients reported significant improvements in back pain and HRQoL. The results should be interpreted in the context of the non-controlled design of this observational study.

**Electronic supplementary material:**

The online version of this article (10.1007/s00223-018-0437-x) contains supplementary material, which is available to authorized users.

## Introduction

Teriparatide (recombinant human parathyroid hormone, Forsteo®) is an osteoanabolic agent that stimulates osteoblastic bone formation to improve bone quality and mass [1]. Teriparatide was first approved in Europe for up to 18 months’ treatment of postmenopausal women with severe osteoporosis. It subsequently received additional approval for the treatment of osteoporosis in men, and for the treatment of osteoporosis associated with glucocorticoid therapy in men and women at risk of fracture. More recently, the European Medicines Agency approved teriparatide treatment for up to 24 months’ duration [2].

The reduction in risk of new fractures during teriparatide treatment in pivotal phase III randomised controlled trials (RCTs) [3–5] has been supported by data from prospective observational studies, including the European Forsteo® Observational Study (EFOS) [6, 7], the Direct Assessment of Non-vertebral fractures in Community Experience (DANCE) study in the USA [8, 9] and a Japanese observational study [10], as well as registry studies [11–13].

The Extended Forsteo® Observational Study (ExFOS) was a non-interventional, prospective, single-cohort, observational study conducted in eight European countries [14] that addressed the need for a large real-life clinical practice study of teriparatide treatment after the update of the teriparatide European label, i.e. the extended treatment duration to 24 months, as well as the newly approved therapeutic indications in the context of osteoporosis treatment guidelines in the participant countries. Here we report the incidence of clinical vertebral and non-vertebral fractures, as well as changes in back pain and health-related quality of life (HRQoL) over 42 months (i.e. up to 24 months of teriparatide treatment and 18 months of follow-up after stopping teriparatide).

## Methods

### Study Design and Patients

The study design and baseline characteristics of the patient population enrolled in ExFOS [14] and the results of the 24-month teriparatide active treatment phase [15] have been reported previously. Briefly, ExFOS was conducted at 111 centres in Croatia, Denmark, France, Greece, Italy, Norway, Slovenia and Sweden. The first patient was enrolled on 24 May 2006 and the last patient completed the study on 3 February 2016. The study population consisted of men and women with osteoporosis who were judged suitable for teriparatide treatment by their physician. Patients were enrolled during routine clinical practice if they were teriparatide-naïve at enrolment. They were prescribed teriparatide (20 µg administered once daily by subcutaneous self-injection) at the baseline visit. Patients were excluded if they were currently being treated with an investigational drug or procedure, or if teriparatide was contraindicated according to the European label [2]. As this was an observational study collecting data in real-life conditions, there were no further restrictions for patient selection and patients were analysed regardless of whether and for how long they took the prescribed medication. Patients gave written informed consent prior to enrolment and could withdraw without consequences at any time. The study was approved by local ethics committees or review boards, depending on local requirements.

The study consisted of two sequential phases: (1) an active treatment phase of up to 24 months, during which patients were treated with teriparatide (which could be discontinued at any time) and (2) a post-treatment follow-up phase after discontinuation of teriparatide. In France and Sweden, teriparatide was reimbursed for 18 months only; in the other six countries, teriparatide was reimbursed for 24 months (in Denmark and Norway, the reimbursement period was extended from 18 to 24 months while the study was ongoing). During and after treatment with teriparatide, patients could be treated with any pharmacological intervention prescribed by their physician for the treatment of osteoporosis.

### Data Collection

All data collection and patient observations occurred within the normal course of clinical care. Data were collected at the baseline visit, at approximately 3, 6, 12, 18 and 24 months after starting teriparatide treatment, and at approximately 6, 12 and 18 months after discontinuing teriparatide treatment. For the statistical analyses, actual patient visit dates were assigned to predefined time intervals given in the statistical analysis plan.

Patient information collected included demographic characteristics, medical history, comorbidities and concomitant medications, lifestyle and risk factors for osteoporosis and falls, bone mineral density (BMD) (when available), osteoporotic fracture history (number, location and approximate date) and previous and current osteoporosis therapies. Physicians also recorded the date teriparatide treatment started and stopped. Patient persistence with teriparatide was assessed at each visit through self-report.

### Fractures

The primary endpoint was the incidence of clinical fractures (a composite of clinical vertebral and non-vertebral fractures). Patients were queried at each visit about the incidence of new clinical fractures; for all fractures, the locations and dates were recorded. A clinical vertebral fracture was identified from the presence of a confirmed radiographic vertebral fracture associated with signs or symptoms suggestive of a vertebral fracture [16], in accordance with the physicians’ clinical practice. Morphometric, asymptomatic vertebral fractures were neither collected nor included in the analyses. Secondary fracture outcomes included non-vertebral fragility fractures and main non-vertebral fragility fractures (forearm/wrist, hip, humerus, leg and ribs).

### Back Pain

Back pain was self-assessed by patients at each visit using a questionnaire evaluating the frequency and severity of back pain as well as limitations of activities and days in bed due to back pain, all during the past month before the visit [6]. Patients also rated the severity of back pain using a visual analogue scale (VAS) ranging from 0 mm (no back pain) to 100 mm (worst possible back pain), which has been shown to be a reliable measure of pain [17]. In addition, patients indicated the type and frequency of analgesic medication used for their back pain in the past month.

### Health-Related Quality of Life

HRQoL was self-assessed by patients at each visit using the EuroQoL-5 Dimension (EQ-5D) questionnaire [18]. Patients rated their current health state in five domains (mobility, self-care, usual activities, pain/discomfort and anxiety/depression), scoring each domain on a 3-point scale. From the domain scores, a single Health State Value (EQ-5D HSV) was calculated based on the UK scoring algorithm [19]. Patients also rated their overall health status on the day of assessment using the EQ-5D VAS (EQ-VAS), which ranged from 0 (worst imaginable health state) to 100 (best imaginable health state).

### Safety

Spontaneously reported adverse events were collected throughout the study.

### Statistical Analysis

Data were analysed for the Total Study Cohort, which—given the pragmatic design of the study—included patients with baseline data and at least one post-baseline visit with non-missing effectiveness data, regardless of whether and for how long they had taken teriparatide after its prescription (i.e. an intention-to-treat principle was applied). Two additional cohorts were predefined and analysed: (1) the Active Treatment Cohort, which included patients with baseline data and at least one visit at or before the end of teriparatide treatment with non-missing data; this excluded patients who did not take teriparatide [15]; (2) the Post-Treatment Follow-Up Cohort, which included patients with baseline data who had received and then discontinued teriparatide, and had at least one post-teriparatide visit with non-missing data; results for this cohort are also reported here. All models used and analyses performed were prespecified in the statistical analysis plan.

Descriptive statistics (frequencies, percentages, means and standard deviations [SDs] or medians with interquartile ranges [Q1, Q3]) were used to describe the study population, persistence with teriparatide treatment and safety results. Incident fractures, back pain and HRQoL were summarised over 42 months. While patients could remain in the study beyond 42 months, most of the reported analyses were restricted to this period because of low patient numbers at later time points.

The number of patients with at least one clinical fracture was summarised in 6-month intervals up to 42 months. Logistic regression analysis with repeated measures was used to assess the respective odds of fracture (i.e. the ratio of patients who had a fracture in an interval vs those who did not have a fracture in that interval). Patients were included in the model at all observed intervals, regardless of whether they had a fracture during the previous interval or were taking teriparatide. The repeated observations of each patient were assumed to be related but no further assumptions were made about the relationship. Contrasts were made between the odds of fracture in the first 6-month period (the reference interval) and each subsequent 6-month period. The results are presented as odds ratios (OR), 95% confidence intervals (95% CI) and *p* values. Fracture modelling was repeated for clinical vertebral fractures, all non-vertebral fractures and the main non-vertebral fractures.

A Cox proportional hazards model was used to evaluate the association of the baseline covariates used in the adjusted logistic regression model with time to first on-study fracture. The results are presented as hazard ratios (HR) with associated 95% CIs.

Changes from baseline in back pain VAS, EQ-5D HSV and EQ-VAS scores were analysed for each visit using mixed models for repeated measures (MMRM), adjusting for preselected variables. For each visit, the numbers of patients reporting an improvement, no change or worsening from baseline in back pain frequency, severity and limitations in activities during the month prior to the visit and in each EQ-5D domain were calculated and analysed using the Wilcoxon signed-rank test.

Statistical analyses were conducted using SAS version 9.4 (SAS Institute, Cary, USA).

## Results

### Patient Disposition and Characteristics

**Fig. 1:**
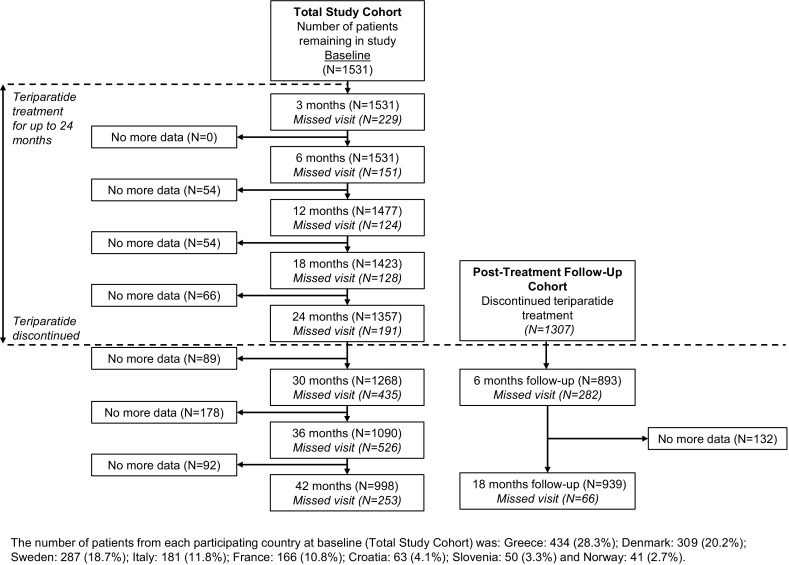
Patient disposition through the study from baseline up to 42 months (Total Study Cohort and Post-Treatment Follow-up Cohort)

Of the 1611 patients enrolled in ExFOS, 1531 were analysed in the Total Study Cohort, including 76 subjects who had a teriparatide prescription but never took the drug. Patient flow through the study and the number of patients at each visit for the Total Study Cohort are presented in Fig. 1. Within this cohort, 998 (65.2%) patients completed 42 months of follow-up.

Baseline characteristics of the Total Study Cohort are summarised in Table 1. Mean (SD) age was 70.3 (9.8) years, the majority (90.7%) were female, 14.6% were taking glucocorticoids and 88.6% reported taking prior osteoporosis medication. At baseline, 85.4% of patients reported at least one previous osteoporotic fracture and 66.2% had two or more previous osteoporotic fractures. Overall, 77.3% of patients had a previous vertebral fracture (54.6% had at least two previous vertebral fractures) and 42.2% had a previous non-vertebral fracture. The median (Q1, Q3) number of previous fractures was 2.0 (1.0, 4.0). A high proportion of patients reported having fractures in the 12 months before baseline: 47.6% had any type of fracture and 41.6% had a vertebral fracture (Table 1).

**Table 1:** Patient baseline characteristics

Characteristic	Total Study Cohort^a^ (*N* = 1531)
Female, *n* (%)	1388 (90.7)
Age, mean (SD) years	70.3 (9.8)
Body mass index, mean (SD) kg/m^2^	25.5 (4.5)
Patients with previous fracture, *n* (%)	1308 (85.4)
Patients with previous vertebral fracture, *n* (%)	1184 (77.3)
Number of previous fractures, median (Q1, Q3)	2.0 (1.0, 4.0)
Number of previous vertebral fractures, median (Q1, Q3)	2.0 (1.0, 3.0)
Patients with fractures in the 12 months before starting teriparatide, *n* (%)	729 (47.6)
Patients with vertebral fractures in the 12 months before starting teriparatide, *n* (%)	637 (41.6)
Maternal history of hip fracture, *n* (%)	248 (19.5)
BMD T-score, mean (SD)	
Lumbar spine (*N* = 1197)^b^	− 3.03 (1.25)
Total hip (*N* = 904)^b^	− 2.44 (1.02)
Femoral neck (*N* = 585)^b^	− 2.51 (0.93)
Uses arms when standing from chair, *n* (%)	795 (52.2)
Sight problems, *n* (%)	492 (32.4)
Current smoker, *n* (%)	222 (14.7)
Exercises ≥ 1 h/week, *n* (%)	907 (60.1)
Has at least one alcoholic drink per week, *n* (%)	533 (35.6)
Number of patients with falls in previous year, *n* (%)	
1 Fall	302 (20.4)
> 1 Fall	276 (18.7)
Immobilised for > 12 months, *n* (%)	48 (3.2)
Reproductive history for females (*N* = 1388)	
Reached menopause, *n* (%)	1044 (98.5)
Years since onset of menopause, median (Q1, Q3)	23.0 (16.0, 30.0)
Early menopause (< 40 years of age), *n* (%)	75 (5.9)
Surgical menopause, *n* (%)	147 (11.1)
Nulliparous, *n* (%)	152 (11.0)
Prior osteoporosis medication, *n* (%)	1357 (88.6)
Prior bisphosphonate use, *n* (%)	989 (64.6)
Duration of prior bisphosphonate therapy (months), mean (SD)	20.8 (37.2)
Current comorbidities, any disease, *n* (%)	519 (33.9)
Rheumatoid arthritis or other rheumatological disorder, *n* (%)	171 (11.3)
Taking glucocorticoids, *n* (%)	224 (14.6)

Percentages are based on patients with non-missing data

^a^Includes all patients with a baseline visit and at least one post-baseline visit, with non-missing effectiveness data

^b^Number of patients with corresponding BMD measurement at baseline

When the main baseline characteristics and risk factors of participants were summarised descriptively by gender, a higher proportion of men than women had previous fracture (91.6 vs. 84.8%), previous vertebral fracture (84.6 vs. 76.6%) and were taking glucocorticoids at baseline (21.7 vs. 13.9%), while fewer men had taken prior osteoporosis medication (76.2 vs. 89.9%).

### Teriparatide Treatment (Active Treatment Cohort)

**Fig. 2:**
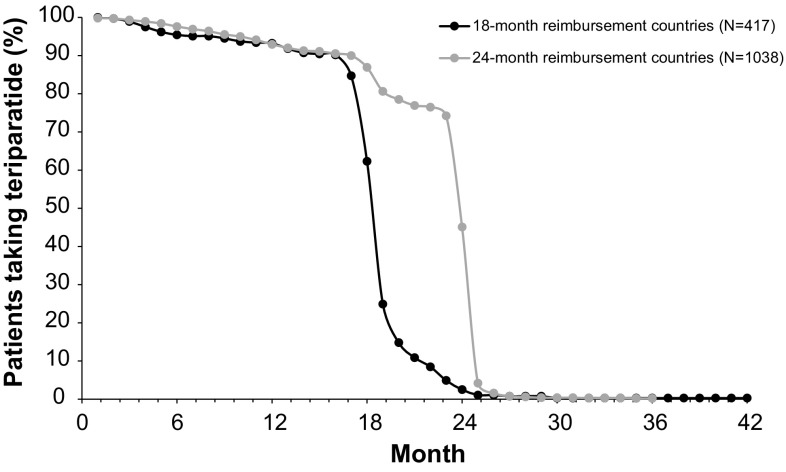
Persistence with teriparatide over time differentiated by 18- and 24-month reimbursement countries (data from Active Treatment Cohort). Countries with 24 months’ reimbursement for teriparatide (Croatia, Denmark, Greece, Italy, Norway, Slovenia) (*n* = 1038). Data missing for 41 patients. Countries with 18 months’ reimbursement for teriparatide (France, Sweden) (*n* = 417). Data missing for 51 patients

The median duration of treatment with teriparatide in the 1454 analysed patients, excluding total treatment interruption (sum of treatment interruption in days from 0‒24 months), was 719 days (23.6 months; Q1; Q3: 18.1; 24.0 months; mean [SD]: 20.7 [5.2] months; range 0–66 months). Treatment interruptions of more than 4 weeks at any time up to 24 months occurred in 67 patients (4.6%). The mean (SD) number of missed injections, calculated as the sum of reported missed injections during the last month before each visit, was 3.9 (8.9). Persistence with teriparatide treatment in the 18- and 24-month reimbursement countries is shown in Fig. 2: 88.6% of all evaluable patients were still taking teriparatide at 17 months.

The majority of patients took calcium (86%) and vitamin D supplementation (98%) during teriparatide treatment.

### Fractures

**Fig. 3:**
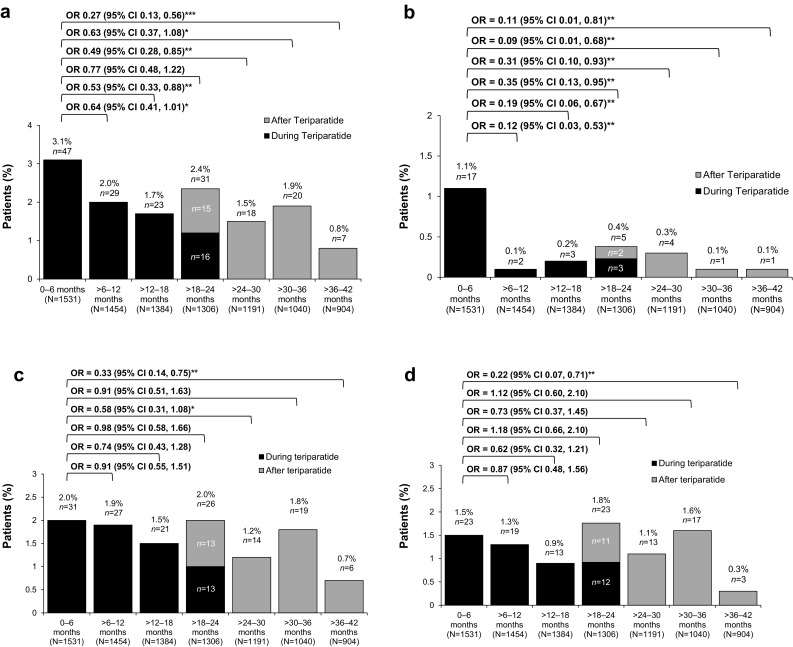
Fracture outcomes in the Total Study Cohort during and after teriparatide treatment for **a** clinical fractures, **b** clinical vertebral fractures, **c** non-vertebral fractures and **d** main non-vertebral fractures (forearm/wrist, hip, humerus, leg or ribs). To reflect real-life outcomes, 76 patients who never started treatment with teriparatide were also included in the analyses. *p < 0.1, **p < 0.05, ***p < 0.001; % and *n* above columns indicate percentage and number of patients at each time point with ≥ 1 fracture. For each patient with a fracture in the > 18- to 24-month interval, we determined whether the fracture occurred while they were still taking teriparatide from the date of fracture and teriparatide stop date. Repeated measures logistic regression model adjusted for time point, age, sex, prior bisphosphonate use and a history of fracture in the 12 months before starting teriparatide; *N* number of patients with information on fractures in the time interval, *CI* confidence interval, *OR* odds ratio

Figure 3a shows the number and percentage of patients with at least one incident clinical fracture in each 6-month interval up to 42 months of follow-up in the Total Study Cohort. The ORs and 95% CIs show a 47% decrease in the adjusted odds of fracture in the > 12- to 18-month period (*p* = 0.013), a 51% decrease in the > 24- to 30-month period (*p* = 0.010) and a 73% decrease in the > 36- to 42-month period (*p* < 0.001) versus the first 6-month period. Note that the > 18- to 24-month period included a mix of subjects who were still taking teriparatide plus those who stopped the drug after 18 months mainly due to the different reimbursement status in the participant countries. The proportions of patients with incident clinical vertebral fractures, non-vertebral fractures and main non-vertebral fractures in each 6-month period up to 42 months are shown in Fig. 3b–d, respectively. Compared with the first 6-month interval, there was a significant reduction in the adjusted odds of clinical vertebral fracture at all subsequent time points (Fig. 3b), but not for non-vertebral fractures. Details of the main non-vertebral fractures by location during the 42 months are included in Online Resource 1.

Excluding the 76 patients who never started therapy with teriparatide from the analyses yielded similar fracture results (data not shown).

For the Total Study Cohort, the hazard of clinical fracture was greater for patients with a history of vertebral and non-vertebral fracture in the 12 months before starting teriparatide versus those without such a history (Table 2). The other covariates in the Cox model (age, gender and prior bisphosphonate use) for time to first clinical fracture were not statistically significant.

**Table 2:** Cox proportional hazards analysis of time to first clinical fracture (Total Study Cohort)

Baseline variable	Comparison	HR (95% CI)
Gender	Female versus male	1.96 (0.96, 4.01)
Age	Unit = 1^a^	1.01 (0.99, 1.03)
Prior bisphosphonate use	Yes versus no	1.35 (0.97, 1.88)
History of vertebral fracture in the last 12 months before starting teriparatide treatment	Yes versus no	1.65 (1.23, 2.23)
History of non-vertebral fracture in the last 12 months before starting teriparatide treatment	Yes versus no	1.82 (1.17, 2.82)

*CI* confidence interval, *HR* hazard ratio

^a^Each additional year of age

### Back Pain

**Fig. 4:**
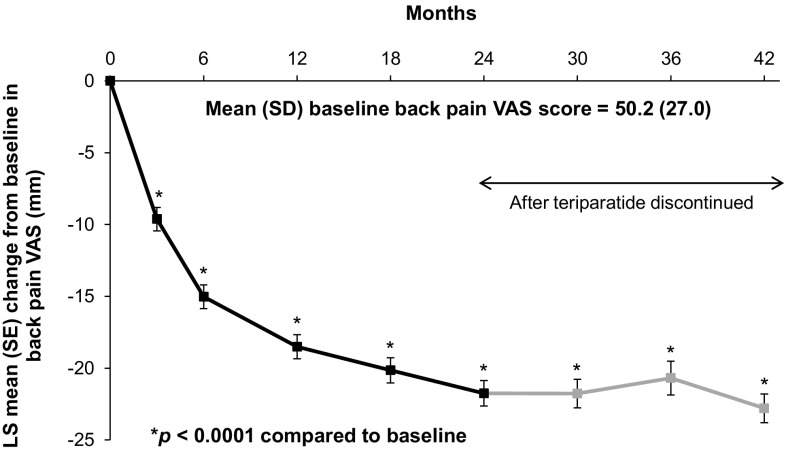
Change in back pain VAS score from baseline. Mixed model for repeated measures (MMRM) included change from baseline in back pain VAS score as a dependent variable, visit as a fixed repeated effect and baseline back pain VAS score, age, duration of prior bisphosphonate therapy, number of vertebral fractures at baseline, vertebral fractures in the 12 months before starting teriparatide treatment and diagnosis of rheumatoid arthritis or other rheumatological disorder as covariates. The unadjusted mean (SD) back pain VAS scores at 3, 6, 12, 18, 24, 30, 36 and 42 months were 41.1 (25.4), 35.6 (24.4), 32.0 (23.9), 30.3 (24.1), 27.8 (24.4), 24.6 (23.5), 31.6 (25.0) and 22.3 (22.2) mm, respectively. *LS* least squares, *SD* standard deviation, *SE* standard error, *VAS* visual analogue scale

The mean (SD) back pain VAS score at baseline was 50.2 (27.0) mm. Figure 4 shows the decrease in the adjusted mean score in back pain VAS score (in mm) from baseline during up to 24 months of teriparatide treatment; this decrease was maintained during the 18 months after teriparatide discontinuation. A sensitivity analysis using an extended adjusted MMRM model that included hours of physical activity at baseline, time since most recent vertebral fracture before baseline and use of analgesics as additional covariates, yielded similar results (see Online Resource 2). Further sensitivity analyses that excluded patients with rheumatoid arthritis also yielded similar results.

Results from the back-pain questionnaire, summarised in Online Resource 3, showed that the frequency and severity of back pain, limitations of activities and days in bed due to back pain in the past month decreased during teriparatide treatment for up to 24 months. This improvement was maintained after teriparatide treatment was discontinued.

The majority of patients reported using analgesic medication for back pain during the month before the baseline assessment (75%; 1104/1470); this number decreased to 64% at 24 months and 63% at 42 months. Among patients who used analgesics, paracetamol was the most commonly used medication at all time points (77% at baseline, 74% at 24 months, 84% at 42 months), followed by acetylsalicylic acid/non-steroidal anti-inflammatory drugs (34% at baseline, 26% at 24 months, 23% at 42 months), low-potency opiates (25% at baseline, 21% at 24 months, 8% at 42 months) and high-potency opiates (11% at baseline, 7% at 24 months, 4% at 42 months).

### Health-Related Quality of Life

**Fig. 5:**
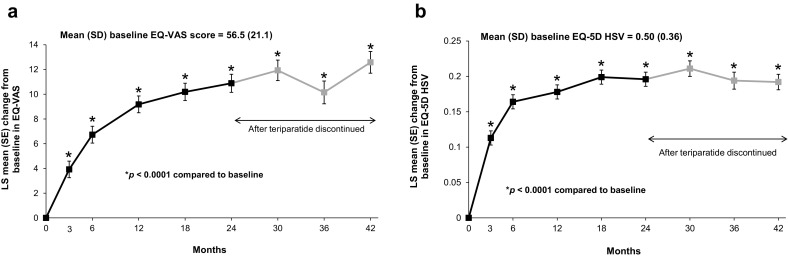
HRQoL changes from baseline for **a** EQ-VAS score and **b** EQ-5D HSV. Mixed models for repeated measures (MMRM) included change from baseline in EQ-VAS score or EQ-5D HSV as a dependent variable, visit as a fixed repeated effect and baseline EQ-VAS score or EQ-5D HSV, age, duration of prior bisphosphonate therapy, number of fractures at baseline (total), fractures in the 12 months before starting teriparatide treatment and diagnosis of rheumatoid arthritis or other rheumatological disorder as covariates. The unadjusted mean (SD) EQ-VAS scores at 3, 6, 12, 18, 24, 30, 36 and 42 months were 61.8 (19.6), 64.8 (19.9), 67.4 (19.6), 68.9 (19.5), 70.1 (20.3), 74.8 (19.3), 67.2 (19.2) and 76.0 (18.5), respectively. The unadjusted mean EQ-5D HSV scores at 3, 6, 12, 18, 24, 30, 36 and 42 months were 0.63 (0.31), 0.68 (0.27), 0.69 (0.28), 0.72 (0.26), 0.73 (0.27), 0.78 (0.24), 0.68 (0.26) and 0.78 (0.24), respectively. *LS* least squares, *SD* standard deviation, *SE* standard error, *EQ-VAS* EuroQol-5 dimension visual analogue scale, *EQ-5D* EuroQol-5 dimension, *HSV* health state value

The mean (SD) EQ-VAS score at baseline was 56.5 (21.1), and the mean (SD) EQ-5D HSV at baseline was 0.50 (0.36). The adjusted mean changes in EQ-VAS score and EQ-5D HSV from baseline during and after teriparatide treatment (Fig. 5) showed statistically significant improvements at all post-baseline time points. The results were similar in the sensitivity analysis using an extended adjusted MMRM model for change from baseline in EQ-VAS score, which included the following additional covariates at baseline: hours of physical activity at baseline, time since most recent fracture (years) before baseline, back pain VAS at baseline, use of analgesics (yes/no) in the month prior to baseline and any comorbidities (see Online Resource 4).

Results for EQ-5D dimensions are summarised in Online Resources 5 and 6; changes over time showed that, at each post-baseline visit, there was a statistically significant improvement compared to baseline. The proportion of patients showing improvement was greatest for the *pain and discomfort* domain (range 25.8% at 3 months to 53.4% at 30 months) and the *usual activities* domain (range 27.8% at 3 months to 47.5% at 42 months) (see Online Resource 6).

### Post-Teriparatide Cohort

Of the 939 patients in the Post-Treatment Follow-Up Cohort with data available, approximately 98% took any osteoporosis medication after teriparatide discontinuation, mainly bisphosphonates (51%). Potent antiresorptives (bisphosphonates, denosumab) were taken by approximately 73% of the cohort. The drugs most commonly taken at least once after stopping teriparatide were denosumab (22%), alendronate and zoledronic acid (20% each). Most patients continued with calcium (80%) and vitamin D supplements (95%) after teriparatide was discontinued. For approximately 24% of the study cohort with data available, calcium and vitamin D was the only osteoporosis treatment taken after teriparatide was discontinued. Details of clinical fractures during the 18 months after stopping teriparatide in the 1307 patients with data are given in Online Resource 7. The risk for new fractures of any category remained very low (< 2% in all 6-month periods analysed), most notably for clinical vertebral fractures (0.2% of patients) and remained stable during the follow-up period.

### Safety

Adverse events experienced by patients in the active treatment phase have already been reported [15]. Of the 1611 enrolled patients, 173 (10.7%) had at least one adverse event and 120 (7.4%) had at least one serious adverse event during the active treatment phase. Of the 339 adverse events reported in this period, 211 (62.2%) were serious and 57 (16.8%) were considered possibly related to study medication. The most common adverse events (> 2%) were fall (7.1%), nausea (4.1%) and headache (2.9%). In the overall Total Study Cohort, which included the post-teriparatide treatment phase, there were a total of 363 adverse events (i.e. 24 more events than during the active treatment phase). No cases of osteosarcoma were observed during the full study duration. There were 38 patients with at least one adverse event leading to death (2.4% of all 1611 enrolled patients); none of these events leading to death were considered related to the study drug by the reporting investigators. In 4 cases, the cause of death was unknown or not reported. As expected in this elderly frail population, the main causes of death were malignancies of different origin (*n* = 7) and vascular acute events (cardiac and cerebral) (*n* = 6), followed by cardiac failure (*n* = 3) and sepsis (*n* = 3). Most of the reported deaths occurred during active teriparatide treatment or shortly after its withdrawal.

## Discussion

The ExFOS study was designed to collect real-world data from European patients with severe osteoporosis in the usual clinical care setting who were prescribed teriparatide for the extended 24 months’ treatment duration and including the latest approved indications. Most of these patients had been widely exposed to prior osteoporosis therapies and included postmenopausal women, men and patients with glucocorticoid-induced osteoporosis. The study also included a post-treatment follow-up observation period of at least 18 months to describe clinical outcomes after teriparatide treatment was discontinued and when patients were treated according to usual clinical care.

As our predefined analysis was based on an intention-to-treat principle, the Total Study Cohort included a small proportion of patients (5%) who received a prescription for teriparatide but never took the drug. This differs from our previous analysis of the 24-month active treatment phase reported by Langdahl et al. [15], where the cohort analysed included only subjects who received at least one dose of teriparatide. Thus, the effectiveness analysis during teriparatide treatment in the current report includes non-compliant subjects and, therefore, more closely reflects real-world clinical practice.

In the Total Study Cohort, the odds of clinical fracture (clinical vertebral plus non-vertebral) were significantly reduced by 47% in the > 12- to 18-month treatment period compared with the first 6 months of treatment, but were not significantly reduced in the > 18- to 24-month interval. One reason for this may be shorter than expected treatment exposure to teriparatide in the > 18- to 24-month period due to lack of reimbursement of the drug after 18 months of treatment in two participant countries (France, Sweden: 29.6% of the Total Study Cohort); this factor was not anticipated when the study was designed. As seen in Fig. 3a, about half of the patients with clinical fractures in the > 18- to 24-month interval no longer took teriparatide. This probably explains the differential finding from the active treatment phase reported by Langdahl et al. [15], where the odds of clinical fracture were significantly reduced by 49% (OR: 0.51, 95% CI 0.29, 0.90; *p* < 0.05) in the > 18- to 24-month interval. Consistent with the cohort analysed by Langdahl et al. [15], however, we observed a significant reduction in the odds of sustaining a clinical vertebral fracture in the > 6- to 12-month, > 12- to 18-month and > 18- to 24-month intervals compared with the first 6-month interval in the Total Study Cohort (Fig. 3b).

The reduction in risk of fracture during teriparatide treatment is consistent with that seen during the observational EFOS study conducted in postmenopausal women with severe osteoporosis who were treated with teriparatide for up to 18 months and were followed up for 18 months after discontinuing teriparatide [20]. Our results extend these findings over a longer treatment period and in a broader range of patients with osteoporosis: most patients in ExFOS were postmenopausal women, 9.3% were men and 14.6% were taking glucocorticoids at baseline.

Our findings also showed that the reduced risk of clinical fracture was maintained even after teriparatide had been discontinued and most patients had switched to another osteoporosis medication. Similarly, in EFOS, the odds of clinical fracture did not change in the 18-month post-teriparatide period, during which 63% of patients took bisphosphonates [7]. The benefits of previous teriparatide therapy were also shown in the follow-up study of the pivotal phase III Fracture Prevention Trial (FPT), which analysed fractures sustained by postmenopausal women with osteoporosis during an observational follow-up period after stopping teriparatide treatment [21]. During 18 months of follow-up, when 47% of patients received osteoporosis medication (mostly bisphosphonates), the risk of new vertebral fractures was significantly reduced by 41% in women who had previously received teriparatide (20 µg/day) relative to the group who had previously received placebo [21].

In contrast to the present study, the DANCE observational study showed a significant reduction in non-vertebral fractures in men and women with osteoporosis treated with teriparatide for up to 24 months [8]. Compared with the first 6-month period, the incidence of new non-vertebral fractures in DANCE was 36, 51 and 43% lower in the 6- to 12-month, 12- to 18-month and 18- to 24-month periods, respectively (all *p* < 0.05) [8]. The differential results between ExFOS and DANCE for the non-vertebral fractures risk reduction may be explained by two important differences between the studies; in DANCE, only patients who received teriparatide were included in the analysis, and the sample size was substantially larger (3720 patients), which may have increased the statistical power to show a reduction in this type of fracture compared to ExFOS.

Osteoporotic fractures are associated with pain, disability and functional limitations that can adversely affect an individual’s quality of life [22–25]. A new vertebral fracture can have long-lasting effects on quality of life for at least 12 months [26], which is why we adjusted for vertebral fractures in the 12 months before starting teriparatide in our analysis of back pain. In the present study, approximately half of the patients had a fracture (42% had a vertebral fracture) in the 12 months before starting teriparatide, and 91% of patients reported having back pain at baseline, which was rated as moderate or severe in most cases. We observed a significant reduction in the severity of patient-reported back pain during up to 24 months of teriparatide treatment, which could also have been influenced by the natural history of pain evolution after a clinical vertebral fracture. Indeed, a post hoc analysis of back pain VAS results by time since last clinical vertebral fracture (data not presented) showed that the baseline mean back pain VAS score was higher in patients with a more recent clinical vertebral fracture (within the last month: 71.1 mm, SD 18.8 mm) than in patients with a clinical vertebral fracture that occurred more than 1 year previously (47.8 mm, SD 26.2 mm). Mean change from baseline back pain VAS score also demonstrated a relationship with the time since the most recent vertebral fracture, with larger decreases in back pain VAS scores in the groups with more recent vertebral fractures.

Although the reduced incidence of new or worsening back pain in teriparatide-treated patients in earlier studies and meta-analyses may have been associated with a reduction in vertebral fractures [27–29], we do not believe this is the reason for our back-pain results in ExFOS, given the very low number of patients with incident clinical vertebral fractures during the course of the study. A similar reduction in back pain VAS scores was seen during 18 months of teriparatide treatment in the EFOS study [7]. Likewise, in the EUROFORS study of postmenopausal women with osteoporosis, teriparatide treatment for 24 months was associated with a significant reduction in back pain [30]. The meta-analysis by Nevitt et al. [29] showed that the reduced risk of developing back pain in teriparatide-treated patients was sustained during 30 months of post-treatment observational follow-up, when 55% of patients received other osteoporosis medication. In ExFOS, we observed a reduction in patient-reported use of strong analgesics (opiates). This is important for patients as it reduces the risk of falls and common side effects, such as nausea, constipation and drowsiness [31, 32].

Consistent with the results on back pain, patients reported a progressive improvement in HRQoL during teriparatide treatment for up to 24 months that was maintained for 18 months after treatment was stopped. Similar HRQoL changes were seen in the EFOS study [33]. Although we observed improvements from baseline in all five domains of the EQ-5D, the largest improvements occurred in the domains of *pain and discomfort* and *usual activities*.

Better long-term persistence with teriparatide has been associated with a lower incidence of fractures [1, 12, 34, 35]. In the current study, although teriparatide was administered by daily injection, persistence was good: 89% of all patients were still taking teriparatide at month 17, while the rate of persistence was 85% at 17 months and 74% at 23 months for countries with an 18- and 24-month reimbursement period, respectively. Moreover, < 5% of patients had treatment gaps of more than 4 weeks during teriparatide treatment.

Most patients (89%) had taken osteoporosis medication before starting teriparatide and 98% took osteoporosis medication after stopping teriparatide. Bisphosphonates were the most commonly used medication, although denosumab was taken by 22% of patients after stopping teriparatide. Because the approved duration of teriparatide treatment is limited to 24 months, subsequent therapy is important to maintain the gains in bone density made with teriparatide [36]. In the FPT follow-up study, discontinuation of teriparatide resulted in a progressive, slow decrease in total hip and femoral neck BMD among patients who took no osteoporosis drug therapy after stopping teriparatide, with BMD values returning to pre-treatment levels approximately 30 months after stopping treatment [37]. However, the optimal sequential therapy after discontinuation of teriparatide remains unclear, and studies in this area have not been powered to measure fracture outcomes but rather use surrogate markers, such as BMD and biochemical markers of bone turnover [38, 39]. Existing evidence supports the use of antiresorptive agents (including bisphosphonates, raloxifene and denosumab) after stopping teriparatide, since use of such drugs can maintain or further increase BMD after teriparatide discontinuation [37–42]. However, fracture outcomes based on properly designed studies of sequential therapy are still lacking.

The real-life results of the ExFOS study are supportive for the role of bone anabolic agents as first-line therapy in patients with severe osteoporosis and prevalent fractures, followed by antiresorptives whenever possible. This therapy sequence is also supported by early reports with teriparatide [37] as well as recent phase III clinical trials of short-term treatment with osteoanabolic agents, such as abaloparatide [43] and romosozumab [44], followed by potent antiresorptives.

The ExFOS study has several potential limitations, the main one being the lack of a non-teriparatide control group. Therefore, we used the first 6-month treatment period as the reference interval for calculating the ORs for fracture, because Kaplan–Meier analysis of non-vertebral fractures in the FPT showed that fracture incidence in the teriparatide and placebo groups began to separate after approximately 9 months of treatment [3]. Although we controlled for prior fracture in the statistical analyses, there may have been some regression to the mean over time, irrespective of treatment. Second, the relatively small number of patients with incident fractures resulted in limited statistical power to detect follow-up differences in non-vertebral fractures compared to the first 6-month reference interval. Likewise, there were too few patients with incident fractures for subgroup analyses, including comparison of fracture rates in patients with and without prior bisphosphonate use at baseline. However, recent subgroup analyses of the fracture data from postmenopausal women with severe osteoporosis in the VERtebral fracture treatment comparisons in Osteoporotic women (VERO) randomised clinical trial showed that the antifracture efficacy of teriparatide, compared with risedronate, did not differ significantly between patients with prior bisphosphate use vs treatment-naïve patients or between patients with and without recent bisphosphonate use [45]. Similarly, the VERO trial found no effect on overall efficacy results of prior recent clinical vertebral fracture, or number and severity of prior vertebral fractures [45]. Third, the shorter duration of teriparatide treatment in France and Sweden due to reimbursement criteria impacted the analysis of 18- to 24-month fracture results. Fourth, persistence with teriparatide was assessed by patient self-report (which may be subject to recall bias) and was not measured quantitatively. Thus, it cannot be ruled out that variability in treatment persistence between patients had an impact on fracture risk analyses, especially considering that we also included in the analysis 5% of patients who never took teriparatide. Finally, there may be a risk of bias due to the preferential drop out of more severely affected patients.

Our study has several strengths. The observational study design permitted inclusion of a broad range of patients with severe osteoporosis (in accordance with the approved European label) and their assessment in a real-world clinical setting. As the study included long-term follow-up after teriparatide was stopped and patients were switched to other osteoporosis therapies, it reflects real-life clinical practice, making the results applicable to the general osteoporosis population in Europe. In addition, our predefined analyses adjusted for variables that could potentially influence the fracture, back pain and HRQoL results, thereby underscoring the robustness of the results.

## Conclusions

In this observational study of postmenopausal women, men and patients with glucocorticoid-induced osteoporosis, patients treated with teriparatide for up to 24 months in routine clinical practice showed a reduction in the incidence of clinical fractures, notably vertebral fractures, which was maintained for at least 18 months after teriparatide was discontinued. This was accompanied by reductions in back pain and analgesic use, and improvement in HRQoL, which were also maintained after treatment discontinuation. Our observations support the use of teriparatide for the treatment of patients with osteoporosis at high risk of fracture for the full-approved treatment duration, followed by antiresorptive drugs. However, our results should be interpreted conservatively in the context of the design of an observational study.

## Electronic supplementary material

Below is the link to the electronic supplementary material.

Supplementary material 1 (PPTX 46 KB)

Supplementary material 2 (PPTX 62 KB)

Supplementary material 3 (PPTX 57 KB)

Supplementary material 4 (PPTX 65 KB)

Supplementary material 5 (PPTX 50 KB)

Supplementary material 6 (PPTX 49 KB)

Supplementary material 7 (PPTX 43 KB)

## References

[1] Lindsay R, Krege JH, Marin F, Jin L, Stepan JJ (2016). Teriparatide for osteoporosis: importance of the full course. Osteoporos Int.

[2] European Medicine Agency Forsteo (teriparatide): EPAR—product information. http://www.ema.europa.eu/docs/en_GB/document_library/EPAR_-_Product_Information/human/000425/WC500027994.pdf. Accessed 6 Jan 2017

[3] Neer RM, Arnaud CD, Zanchetta JR, Prince R, Gaich GA, Reginster JY, Hodsman AB, Eriksen EF, Ish-Shalom S, Genant HK, Wang O, Mitlak BH (2001). Effect of parathyroid hormone (1–34) on fractures and bone mineral density in postmenopausal women with osteoporosis. N Engl J Med.

[4] Kaufman JM, Orwoll E, Goemaere S, San Martin J, Hossain A, Dalsky GP, Lindsay R, Mitlak BH (2005). Teriparatide effects on vertebral fractures and bone mineral density in men with osteoporosis: treatment and discontinuation of therapy. Osteoporos Int.

[5] Saag KG, Shane E, Boonen S, Marín F, Donley DW, Taylor KA, Dalsky GP, Marcus R (2007). Teriparatide or alendronate in glucocorticoid-induced osteoporosis. N Engl J Med.

[6] Langdahl BL, Rajzbaum G, Jakob F, Karras D, Ljunggren O, Lems WF, Fahrleitner-Pammer A, Walsh JB, Barker C, Kutahov A, Marin F (2009). Reduction in fracture rate and back pain and increased quality of life in postmenopausal women treated with teriparatide: 18-month data from the European Forsteo Observational Study (EFOS). Calcif Tissue Int.

[7] Fahrleitner-Pammer A, Langdahl BL, Marin F, Jakob F, Karras D, Barrett A, Ljunggren Ö, Walsh JB, Rajzbaum G, Barker C, Lems WF (2011). Fracture rate and back pain during and after discontinuation of teriparatide: 36-month data from the European Forsteo Observational Study (EFOS). Osteoporos Int.

[8] Silverman S, Miller P, Sebba A, Weitz M, Wan X, Alam J, Masica D, Taylor KA, Ruff VA, Krohn K (2013). The Direct Assessment of Nonvertebral Fractures in Community Experience (DANCE) study: 2-year nonvertebral fragility fracture results. Osteoporos Int.

[9] Beall DP, Feldman RG, Gordon ML, Gruber BL, Lane JM, Valenzuela G, Yim D, Alam J, Krege JH, Krohn K (2016). Patients with prior vertebral or hip fractures treated with teriparatide in the Direct Assessment of Nonvertebral Fractures in Community Experience (DANCE) observational study. Osteoporos Int.

[10] Yamamoto T, Taketsuna M, Guo X, Sato M, Sowa H (2014). The safety and effectiveness profile of daily teriparatide in a prospective observational study in Japanese patients with osteoporosis at high risk for fracture: interim report. J Bone Miner Metab.

[11] Boytsov N, Zhang X, Sugihara T, Taylor K, Swindle R (1996). Osteoporotic fractures and associated hospitalizations among patients treated with teriparatide compared to a matched cohort of patients not treated with teriparatide. Curr Med Res Opin.

[12] Burge R, Sato M, Sugihara T (2016). Real-world clinical and economic outcomes for daily teriparatide patients in Japan. J Bone Miner Metab.

[13] Burge RT, Disch DP, Gelwicks S, Zhang X, Krege JH (2017). Hip and other fragility fracture incidence in real-world teriparatide-treated patients in the United States. Osteoporosis Int.

[14] Ljunggren O, Benhamou CL, Dekker J, Kapetanos G, Kocjan T, Langdahl BL, Napoli N, Petto H, Nikolic T, Lindh E (2014). Study description and baseline characteristics of the population enrolled in a multinational observational study of extended teriparatide use (ExFOS). Curr Med Res Opin.

[15] Langdahl BL, Ljunggren Ö, Benhamou CL, Marin F, Kapetanos G, Kocjan T, Lespessailles E, Napoli N, Nikolic T, Petto H, Moll T, Lindh E (2016). Fracture rate, quality of life and back pain in patients with osteoporosis treated with teriparatide: 24-month results from the Extended Forsteo Observational Study (ExFOS). Calcif Tissue Int.

[16] Ross PD (1997). Clinical consequences of vertebral fractures. Am J Med.

[17] Sriwatanakul K, Kelvie W, Lasagna L, Calimlim JF, Weis OF, Mehta G (1983). Studies with different types of visual analog scales for measurement of pain. Clin Pharmacol Ther.

[18] Brooks R (1996). EuroQol: the current state of play. Health Policy.

[19] Szende A, Williams A (2004) Measuring self-reported health: an international perspective based on EQ-5D. EuroQol, Rotterdam. http://www.euroqol.org/fileadmin/user_upload/Documenten/PDF/Books/Measuring_Self-Reported_Population_Health_-_An_International_Perspective_based_on_EQ-5D.pdf. Accessed 26 Sept 2013

[20] Rajzbaum G, Jakob F, Karras D, Ljunggren O, Lems WF, Langdahl BL, Fahrleitner-Pammer A, Walsh JB, Gibson A, Tynan AJ, Marin F (2008). Characterization of patients in the European Forsteo Observational Study (EFOS): postmenopausal women entering teriparatide treatment in a community setting. Curr Med Res Opin.

[21] Lindsay R, Scheele WH, Neer R, Pohl G, Adami S, Mautalen C, Reginster J-Y, Stepan JJ, Myers SL, Mitlak BH (2004). Sustained vertebral fracture risk reduction after withdrawal of teriparatide in postmenopausal women with osteoporosis. Arch Intern Med.

[22] Hallberg I, Rosenqvist AM, Kartous L, Löfman O, Wahlström O, Toss G (2004). Health-related quality of life after osteoporotic fractures. Osteoporos Int.

[23] Silverman SL, Piziak VK, Chen P, Misurski DA, Wagman RB (2005). Relationship of health-related quality of life to prevalent and new or worsening back pain in postmenopausal women with osteoporosis. J Rheumatol.

[24] Francis RM, Aspray TJ, Hide G, Sutcliffe AM, Wilkinson P (2008). Back pain in osteoporotic vertebral fractures. Osteoporos Int.

[25] Wilson S, Sharp CA, Davie MW (2012). Health-related quality of life in patients with osteoporosis in the absence of vertebral fracture: a systematic review. Osteoporos Int.

[26] Suzuki N, Ogikubo O, Hansson T (2008). The course of the acute vertebral body fragility fracture: its effect on pain, disability and quality of life during 12 months. Eur Spine J.

[27] Genant HK, Halse J, Briney WG, Xie L, Glass EV, Krege JH (2005). The effects of teriparatide on the incidence of back pain in postmenopausal women with osteoporosis. Curr Med Res Opin.

[28] Nevitt MC, Chen P, Dore RK, Reginster JY, Kiel DP, Zanchetta JR, Glass EV, Krege JH (2006). Reduced risk of back pain following teriparatide treatment: a meta-analysis. Osteoporos Int.

[29] Nevitt MC, Chen P, Kiel DP, Reginster J-Y, Dore RK, Zanchetta JR, Glass EV, Krege JH (2006). Reduction in the risk of developing back pain persists at least 30 months after discontinuation of teriparatide treatment: a meta-analysis. Osteoporos Int.

[30] Lyritis G, Marin F, Barker C, Pfeifer M, Farrerons J, Brixen K, del Pino J, Keen R, Nickelsen TN, EUROFORS Study Group (2010). Back pain during different sequential treatment regimens of teriparatide: results from EUROFORS. Curr Med Res Opin.

[31] Vellucci R, Mediati RD, Ballerini G (2014). Use of opioids for treatment of osteoporotic pain. Clin Cases Miner Bone Metab.

[32] Vestergaard P (2008). Pain-relief medication and risk of fractures. Curr Drug Saf.

[33] Ljunggren Ö, Barrett A, Stoykov I, Langdahl BL, Lems WF, Walsh JB, Fahrleitner-Pammer A, Rajzbaum G, Jakob F, Karras D, Marin F (2013). Effective osteoporosis treatment with teriparatide is associated with enhanced quality of life in postmenopausal women with osteoporosis: the European Forsteo Observational Study. BMC Musculoskelet Disord.

[34] Lindsay R, Miller P, Pohl G, Glass EV, Chen P, Krege JH (2009). Relationship between duration of teriparatide therapy and clinical outcomes in postmenopausal women with osteoporosis. Osteoporos Int.

[35] Yu S, Burge RT, Foster SA, Gelwicks S, Meadows ES (2012). The impact of teriparatide adherence and persistence on fracture outcomes. Osteoporos Int.

[36] Bilezikian JP, Rubin MR (2006). Combination/sequential therapies for anabolic and antiresorptive skeletal agents for osteoporosis. Curr Osteoporos Rep.

[37] Prince R, Sipos A, Hossain A, Syversen U, Ish-Shalom S, Marcinowska E, Halse J, Lindsay R, Dalsky GP, Mitlak BH (2005). Sustained nonvertebral fragility fracture risk reduction after discontinuation of teriparatide treatment. J Bone Miner Res.

[38] Cosman F (2014). Anabolic and antiresorptive therapy for osteoporosis: combination and sequential approaches. Curr Osteoporos Rep.

[39] Cosman F, Nieves JW, Dempster DW (2017). Treatment sequence matters: anabolic and antiresorptive therapy for osteoporosis. J Bone Miner Res.

[40] Kurland ES, Heller SL, Diamond B, McMahon DJ, Cosman F, Bilezikian JP (2004). The importance of bisphosphonate therapy in maintaining bone mass in men after therapy with teriparatide [human parathyroid hormone (1–34)]. Osteoporos Int.

[41] Eastell R, Nickelsen T, Marin F, Barker C, Hadji P, Farrerons J, Audran M, Boonen S, Brixen K, Gomes JM, Obermayer-Pietsch B, Avramidis A, Sigurdsson G, Glüer CC (2009). Sequential treatment of severe postmenopausal osteoporosis after teriparatide: final results of the randomized, controlled European Study of Forsteo (EUROFORS). J Bone Miner Res.

[42] Lou S, Lv H, Wang G, Li Z, Li M, Zhang L, Tang P (2016). The effect of sequential therapy for postmenopausal women with osteoporosis. A PRISMA-compliant meta-analysis of randomized controlled trials. Medicine (Baltimore).

[43] Cosman F, Miller PD, Williams GC, Hattersley G, Hu MY, Valter I, Fitzpatrick LA, Riis BJ, Christiansen C, Bilezikian JP, Black D (2017). Eighteen months of treatment with subcutaneous abaloparatide followed by 6 months of treatment with alendronate in postmenopausal women with osteoporosis: results of the ACTIVExtend trial. Mayo Clin Proc.

[44] Cosman F, Crittenden DB, Adachi JD, Binkley N, Czerwinski E, Ferrari S, Hofbauer LC, Lau E, Lewiecki EM, Miyauchi A, Zerbini CA, Milmont CE, Chen L, Maddox J, Meisner PD, Libanati C, Grauer A (2016). Romosozumab treatment in postmenopausal women with osteoporosis. N Engl J Med.

[45] Geusens P, Marin F, Kendler DL, Russo LA, Zerbini CA, Minisola S, Body JJ, Lespessailles E, Greenspan SL, Bagur A, Stepan JJ, Lakatos P, Casado E, Moericke R, López-Romero P, Fahrleitner-Pammer A (2018). Effects of teriparatide compared with risedronate on the risk of fractures in subgroups of postmenopausal women with severe osteoporosis: the VERO trial. J Bone Miner Res.

