# Supplementary material for: Effects of Teriparatide in Patients with Osteoporosis in Clinical Practice: 42-Month Results During and After Discontinuation of Treatment from the European Extended Forsteo® Observational Study (ExFOS)
Source: Calcif Tissue Int. 2018 Jun 16;103(4):359–71. doi: 10.1007/s00223-018-0437-x (PMC6153867; doi:10.1007/s00223-018-0437-x)
Supplement: Supplementary file 1 — Supplementary material 1 (PPTX 46 KB) [file 223_2018_437_MOESM1_ESM.pptx]

## Slide 1
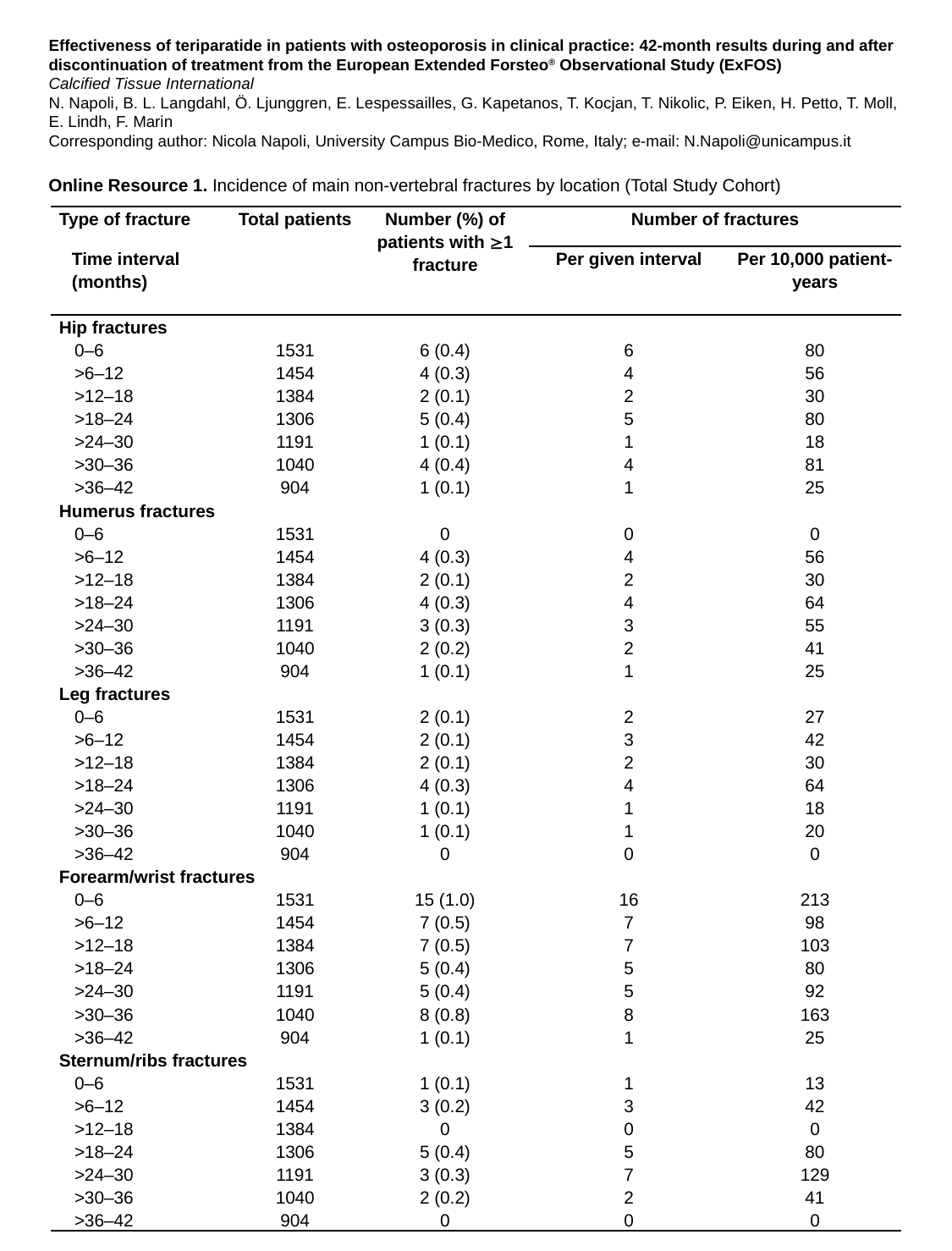

Effectiveness of teriparatide in patients with osteoporosis in clinical practice: 42-month results during and after discontinuation of treatment from the European Extended Forsteo® Observational Study (ExFOS)
Calcified Tissue International
N. Napoli, B. L. Langdahl, Ö. Ljunggren, E. Lespessailles, G. Kapetanos, T. Kocjan, T. Nikolic, P. Eiken, H. Petto, T. Moll, E. Lindh, F. Marin
Corresponding author: Nicola Napoli, University Campus Bio-Medico, Rome, Italy; e-mail: N.Napoli@unicampus.it
Online Resource 1. Incidence of main non-vertebral fractures by location (Total Study Cohort)
| Type of fracture | Total patients | Number (%) of patients with ≥1 fracture | Number of fractures | |
| --- | --- | --- | --- | --- |
| Time interval (months) | | | Per given interval | Per 10,000 patient-years |
| Hip fractures | | | | |
| 0‒6 | 1531 | 6 (0.4) | 6 | 80 |
| >6‒12 | 1454 | 4 (0.3) | 4 | 56 |
| >12‒18 | 1384 | 2 (0.1) | 2 | 30 |
| >18‒24 | 1306 | 5 (0.4) | 5 | 80 |
| >24‒30 | 1191 | 1 (0.1) | 1 | 18 |
| >30‒36 | 1040 | 4 (0.4) | 4 | 81 |
| >36‒42 | 904 | 1 (0.1) | 1 | 25 |
| Humerus fractures | | | | |
| 0‒6 | 1531 | 0 | 0 | 0 |
| >6‒12 | 1454 | 4 (0.3) | 4 | 56 |
| >12‒18 | 1384 | 2 (0.1) | 2 | 30 |
| >18‒24 | 1306 | 4 (0.3) | 4 | 64 |
| >24‒30 | 1191 | 3 (0.3) | 3 | 55 |
| >30‒36 | 1040 | 2 (0.2) | 2 | 41 |
| >36‒42 | 904 | 1 (0.1) | 1 | 25 |
| Leg fractures | | | | |
| 0‒6 | 1531 | 2 (0.1) | 2 | 27 |
| >6‒12 | 1454 | 2 (0.1) | 3 | 42 |
| >12‒18 | 1384 | 2 (0.1) | 2 | 30 |
| >18‒24 | 1306 | 4 (0.3) | 4 | 64 |
| >24‒30 | 1191 | 1 (0.1) | 1 | 18 |
| >30‒36 | 1040 | 1 (0.1) | 1 | 20 |
| >36‒42 | 904 | 0 | 0 | 0 |
| Forearm/wrist fractures | | | | |
| 0‒6 | 1531 | 15 (1.0) | 16 | 213 |
| >6‒12 | 1454 | 7 (0.5) | 7 | 98 |
| >12‒18 | 1384 | 7 (0.5) | 7 | 103 |
| >18‒24 | 1306 | 5 (0.4) | 5 | 80 |
| >24‒30 | 1191 | 5 (0.4) | 5 | 92 |
| >30‒36 | 1040 | 8 (0.8) | 8 | 163 |
| >36‒42 | 904 | 1 (0.1) | 1 | 25 |
| Sternum/ribs fractures | | | | |
| 0‒6 | 1531 | 1 (0.1) | 1 | 13 |
| >6‒12 | 1454 | 3 (0.2) | 3 | 42 |
| >12‒18 | 1384 | 0 | 0 | 0 |
| >18‒24 | 1306 | 5 (0.4) | 5 | 80 |
| >24‒30 | 1191 | 3 (0.3) | 7 | 129 |
| >30‒36 | 1040 | 2 (0.2) | 2 | 41 |
| >36‒42 | 904 | 0 | 0 | 0 |
