# Supplementary material for: Effects of Teriparatide in Patients with Osteoporosis in Clinical Practice: 42-Month Results During and After Discontinuation of Treatment from the European Extended Forsteo® Observational Study (ExFOS)
Source: Calcif Tissue Int. 2018 Jun 16;103(4):359–71. doi: 10.1007/s00223-018-0437-x (PMC6153867; doi:10.1007/s00223-018-0437-x)
Supplement: Supplementary file 2 — Supplementary material 2 (PPTX 62 KB) [file 223_2018_437_MOESM2_ESM.pptx]

## Slide 1
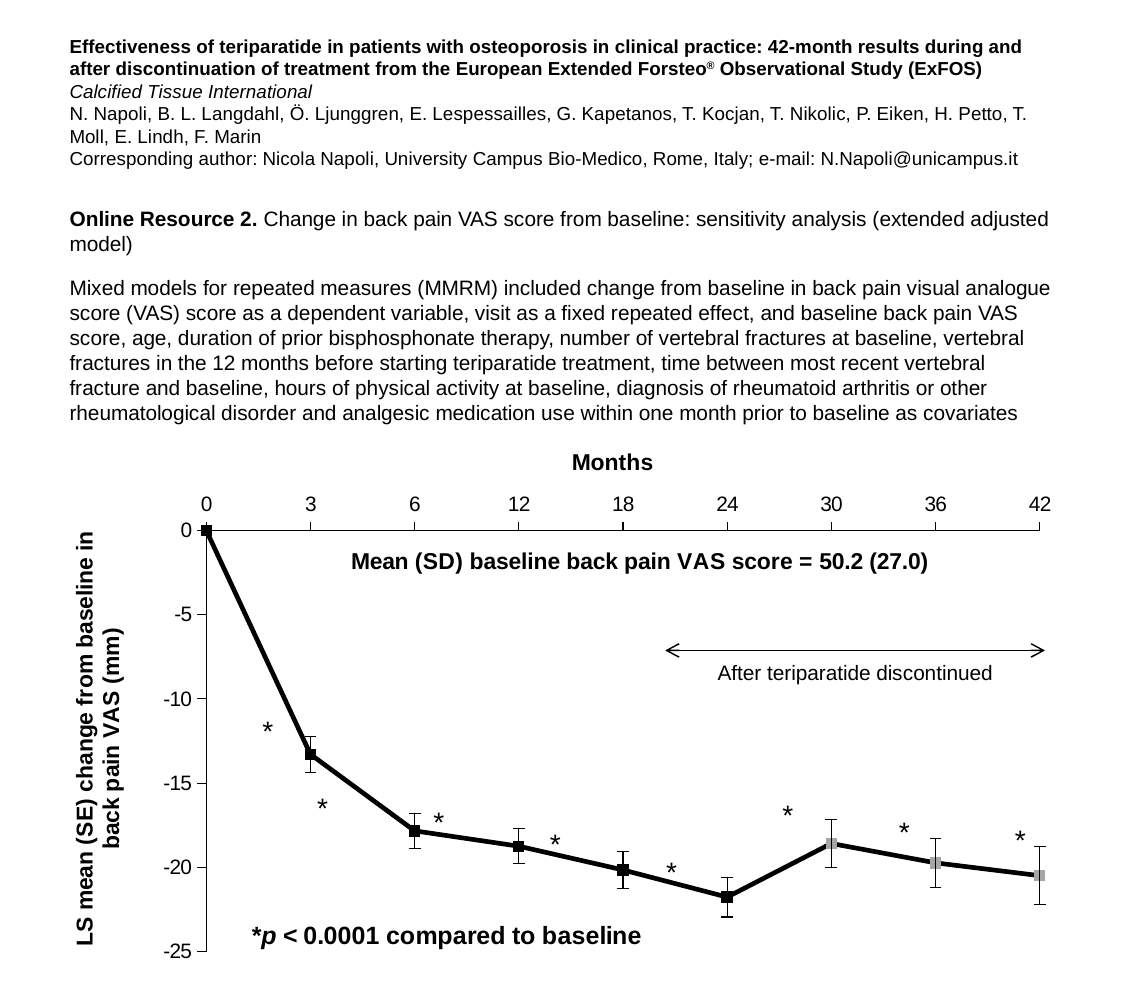

Effectiveness of teriparatide in patients with osteoporosis in clinical practice: 42-month results during and after discontinuation of treatment from the European Extended Forsteo® Observational Study (ExFOS)
Calcified Tissue International
N. Napoli, B. L. Langdahl, Ö. Ljunggren, E. Lespessailles, G. Kapetanos, T. Kocjan, T. Nikolic, P. Eiken, H. Petto, T. Moll, E. Lindh, F. Marin
Corresponding author: Nicola Napoli, University Campus Bio-Medico, Rome, Italy; e-mail: N.Napoli@unicampus.it
Online Resource 2. Change in back pain VAS score from baseline: sensitivity analysis (extended adjusted model)
Mixed models for repeated measures (MMRM) included change from baseline in back pain visual analogue score (VAS) score as a dependent variable, visit as a fixed repeated effect, and baseline back pain VAS score, age, duration of prior bisphosphonate therapy, number of vertebral fractures at baseline, vertebral fractures in the 12 months before starting teriparatide treatment, time between most recent vertebral fracture and baseline, hours of physical activity at baseline, diagnosis of rheumatoid arthritis or other rheumatological disorder and analgesic medication use within one month prior to baseline as covariates
### Chart
| Category | LS mean change from baseline |
|---|---|
| 0 | 0.0 |
| 3 | -13.29 |
| 6 | -17.82999999999999 |
| 12 | -18.73999999999999 |
| 18 | -20.15 |
| 24 | -21.76 |
| 30 | -18.57999999999999 |
| 36 | -19.73 |
| 42 | -20.49 |*
*
*
*
*
*
*
*
After teriparatide discontinued
