# Supplementary material for: Effects of Teriparatide in Patients with Osteoporosis in Clinical Practice: 42-Month Results During and After Discontinuation of Treatment from the European Extended Forsteo® Observational Study (ExFOS)
Source: Calcif Tissue Int. 2018 Jun 16;103(4):359–71. doi: 10.1007/s00223-018-0437-x (PMC6153867; doi:10.1007/s00223-018-0437-x)
Supplement: Supplementary file 3 — Supplementary material 3 (PPTX 57 KB) [file 223_2018_437_MOESM3_ESM.pptx]

## Slide 1
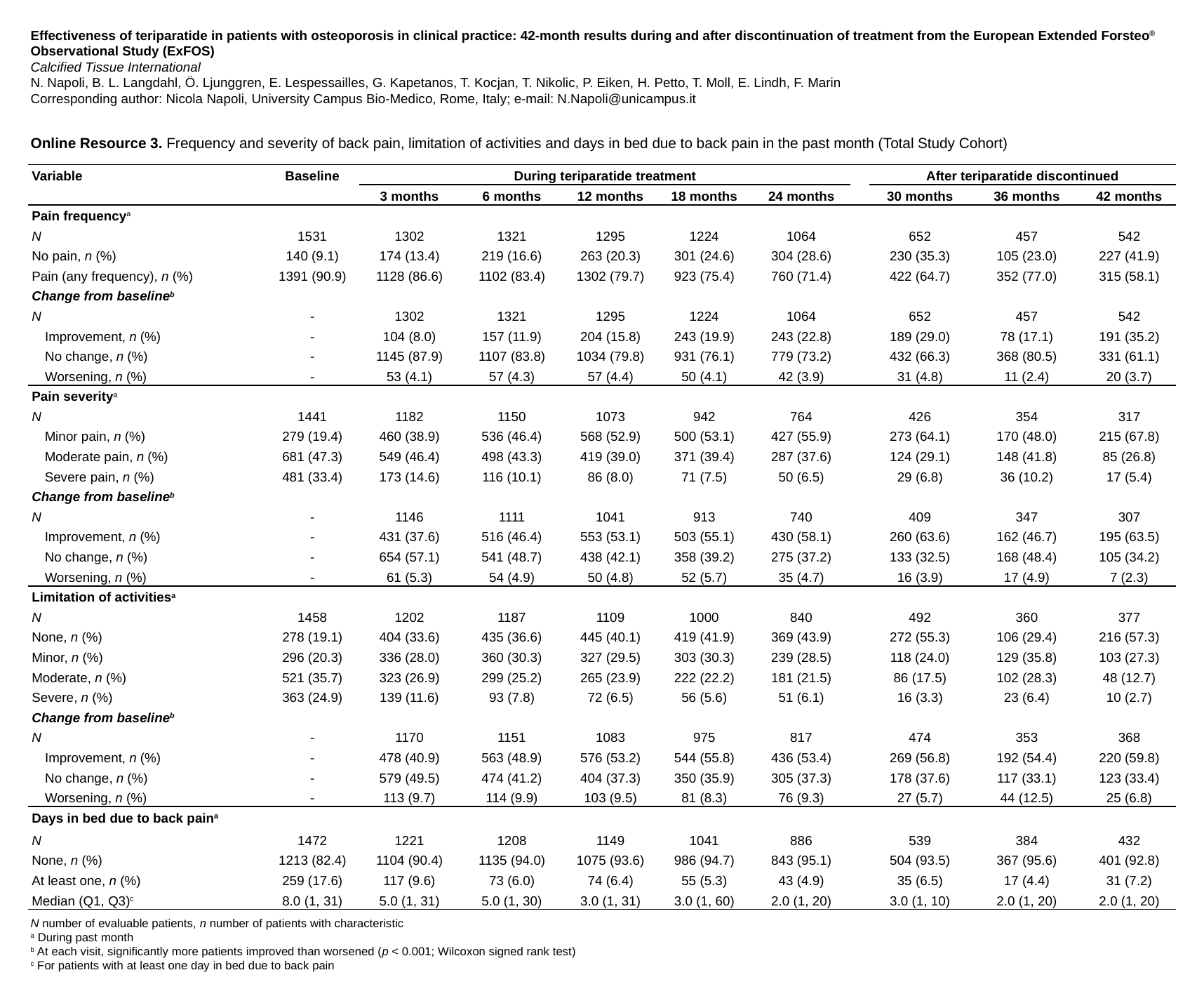

Effectiveness of teriparatide in patients with osteoporosis in clinical practice: 42-month results during and after discontinuation of treatment from the European Extended Forsteo® Observational Study (ExFOS)
Calcified Tissue International
N. Napoli, B. L. Langdahl, Ö. Ljunggren, E. Lespessailles, G. Kapetanos, T. Kocjan, T. Nikolic, P. Eiken, H. Petto, T. Moll, E. Lindh, F. Marin
Corresponding author: Nicola Napoli, University Campus Bio-Medico, Rome, Italy; e-mail: N.Napoli@unicampus.it
Online Resource 3. Frequency and severity of back pain, limitation of activities and days in bed due to back pain in the past month (Total Study Cohort)
| Variable | Baseline | During teriparatide treatment | | | | | | After teriparatide discontinued | | |
| --- | --- | --- | --- | --- | --- | --- | --- | --- | --- | --- |
| | | 3 months | 6 months | 12 months | 18 months | 24 months | | 30 months | 36 months | 42 months |
| Pain frequencya | | | | | | | | | | |
| N | 1531 | 1302 | 1321 | 1295 | 1224 | 1064 | | 652 | 457 | 542 |
| No pain, n (%) | 140 (9.1) | 174 (13.4) | 219 (16.6) | 263 (20.3) | 301 (24.6) | 304 (28.6) | | 230 (35.3) | 105 (23.0) | 227 (41.9) |
| Pain (any frequency), n (%) | 1391 (90.9) | 1128 (86.6) | 1102 (83.4) | 1302 (79.7) | 923 (75.4) | 760 (71.4) | | 422 (64.7) | 352 (77.0) | 315 (58.1) |
| Change from baselineb | | | | | | | | | | |
| N | - | 1302 | 1321 | 1295 | 1224 | 1064 | | 652 | 457 | 542 |
| Improvement, n (%) | - | 104 (8.0) | 157 (11.9) | 204 (15.8) | 243 (19.9) | 243 (22.8) | | 189 (29.0) | 78 (17.1) | 191 (35.2) |
| No change, n (%) | - | 1145 (87.9) | 1107 (83.8) | 1034 (79.8) | 931 (76.1) | 779 (73.2) | | 432 (66.3) | 368 (80.5) | 331 (61.1) |
| Worsening, n (%) | - | 53 (4.1) | 57 (4.3) | 57 (4.4) | 50 (4.1) | 42 (3.9) | | 31 (4.8) | 11 (2.4) | 20 (3.7) |
| Pain severitya | | | | | | | | | | |
| N | 1441 | 1182 | 1150 | 1073 | 942 | 764 | | 426 | 354 | 317 |
| Minor pain, n (%) | 279 (19.4) | 460 (38.9) | 536 (46.4) | 568 (52.9) | 500 (53.1) | 427 (55.9) | | 273 (64.1) | 170 (48.0) | 215 (67.8) |
| Moderate pain, n (%) | 681 (47.3) | 549 (46.4) | 498 (43.3) | 419 (39.0) | 371 (39.4) | 287 (37.6) | | 124 (29.1) | 148 (41.8) | 85 (26.8) |
| Severe pain, n (%) | 481 (33.4) | 173 (14.6) | 116 (10.1) | 86 (8.0) | 71 (7.5) | 50 (6.5) | | 29 (6.8) | 36 (10.2) | 17 (5.4) |
| Change from baselineb | | | | | | | | | | |
| N | - | 1146 | 1111 | 1041 | 913 | 740 | | 409 | 347 | 307 |
| Improvement, n (%) | - | 431 (37.6) | 516 (46.4) | 553 (53.1) | 503 (55.1) | 430 (58.1) | | 260 (63.6) | 162 (46.7) | 195 (63.5) |
| No change, n (%) | - | 654 (57.1) | 541 (48.7) | 438 (42.1) | 358 (39.2) | 275 (37.2) | | 133 (32.5) | 168 (48.4) | 105 (34.2) |
| Worsening, n (%) | - | 61 (5.3) | 54 (4.9) | 50 (4.8) | 52 (5.7) | 35 (4.7) | | 16 (3.9) | 17 (4.9) | 7 (2.3) |
| Limitation of activitiesa | | | | | | | | | | |
| N | 1458 | 1202 | 1187 | 1109 | 1000 | 840 | | 492 | 360 | 377 |
| None, n (%) | 278 (19.1) | 404 (33.6) | 435 (36.6) | 445 (40.1) | 419 (41.9) | 369 (43.9) | | 272 (55.3) | 106 (29.4) | 216 (57.3) |
| Minor, n (%) | 296 (20.3) | 336 (28.0) | 360 (30.3) | 327 (29.5) | 303 (30.3) | 239 (28.5) | | 118 (24.0) | 129 (35.8) | 103 (27.3) |
| Moderate, n (%) | 521 (35.7) | 323 (26.9) | 299 (25.2) | 265 (23.9) | 222 (22.2) | 181 (21.5) | | 86 (17.5) | 102 (28.3) | 48 (12.7) |
| Severe, n (%) | 363 (24.9) | 139 (11.6) | 93 (7.8) | 72 (6.5) | 56 (5.6) | 51 (6.1) | | 16 (3.3) | 23 (6.4) | 10 (2.7) |
| Change from baselineb | | | | | | | | | | |
| N | - | 1170 | 1151 | 1083 | 975 | 817 | | 474 | 353 | 368 |
| Improvement, n (%) | - | 478 (40.9) | 563 (48.9) | 576 (53.2) | 544 (55.8) | 436 (53.4) | | 269 (56.8) | 192 (54.4) | 220 (59.8) |
| No change, n (%) | - | 579 (49.5) | 474 (41.2) | 404 (37.3) | 350 (35.9) | 305 (37.3) | | 178 (37.6) | 117 (33.1) | 123 (33.4) |
| Worsening, n (%) | - | 113 (9.7) | 114 (9.9) | 103 (9.5) | 81 (8.3) | 76 (9.3) | | 27 (5.7) | 44 (12.5) | 25 (6.8) |
| Days in bed due to back paina | | | | | | | | | | |
| N | 1472 | 1221 | 1208 | 1149 | 1041 | 886 | | 539 | 384 | 432 |
| None, n (%) | 1213 (82.4) | 1104 (90.4) | 1135 (94.0) | 1075 (93.6) | 986 (94.7) | 843 (95.1) | | 504 (93.5) | 367 (95.6) | 401 (92.8) |
| At least one, n (%) | 259 (17.6) | 117 (9.6) | 73 (6.0) | 74 (6.4) | 55 (5.3) | 43 (4.9) | | 35 (6.5) | 17 (4.4) | 31 (7.2) |
| Median (Q1, Q3)c | 8.0 (1, 31) | 5.0 (1, 31) | 5.0 (1, 30) | 3.0 (1, 31) | 3.0 (1, 60) | 2.0 (1, 20) | | 3.0 (1, 10) | 2.0 (1, 20) | 2.0 (1, 20) |
N number of evaluable patients, n number of patients with characteristic
a During past month
b At each visit, significantly more patients improved than worsened (p < 0.001; Wilcoxon signed rank test)
c For patients with at least one day in bed due to back pain
