# Supplementary material for: Effects of Teriparatide in Patients with Osteoporosis in Clinical Practice: 42-Month Results During and After Discontinuation of Treatment from the European Extended Forsteo® Observational Study (ExFOS)
Source: Calcif Tissue Int. 2018 Jun 16;103(4):359–71. doi: 10.1007/s00223-018-0437-x (PMC6153867; doi:10.1007/s00223-018-0437-x)
Supplement: Supplementary file 4 — Supplementary material 4 (PPTX 65 KB) [file 223_2018_437_MOESM4_ESM.pptx]

## Slide 1
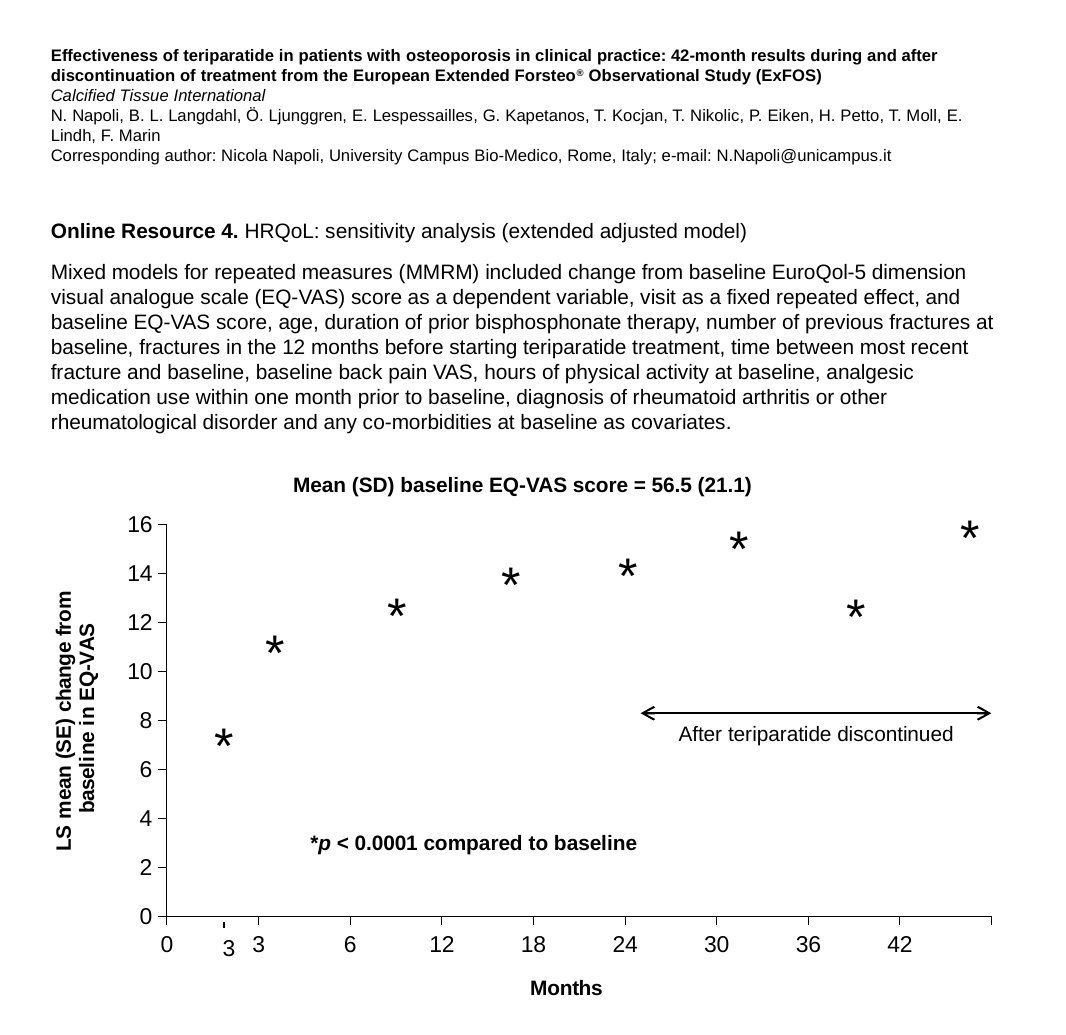

Effectiveness of teriparatide in patients with osteoporosis in clinical practice: 42-month results during and after discontinuation of treatment from the European Extended Forsteo® Observational Study (ExFOS)
Calcified Tissue International
N. Napoli, B. L. Langdahl, Ö. Ljunggren, E. Lespessailles, G. Kapetanos, T. Kocjan, T. Nikolic, P. Eiken, H. Petto, T. Moll, E. Lindh, F. Marin
Corresponding author: Nicola Napoli, University Campus Bio-Medico, Rome, Italy; e-mail: N.Napoli@unicampus.it
Online Resource 4. HRQoL: sensitivity analysis (extended adjusted model)
Mixed models for repeated measures (MMRM) included change from baseline EuroQol-5 dimension visual analogue scale (EQ-VAS) score as a dependent variable, visit as a fixed repeated effect, and baseline EQ-VAS score, age, duration of prior bisphosphonate therapy, number of previous fractures at baseline, fractures in the 12 months before starting teriparatide treatment, time between most recent fracture and baseline, baseline back pain VAS, hours of physical activity at baseline, analgesic medication use within one month prior to baseline, diagnosis of rheumatoid arthritis or other rheumatological disorder and any co-morbidities at baseline as covariates.
Mean (SD) baseline EQ-VAS score = 56.5 (21.1)
### Chart
| Category | LS mean change from baseline |
|---|---|
| 0 | 0.0 |
| 3 | 5.81 |
| 6 | 9.34 |
| 12 | 10.78 |
| 18 | 12.32 |
| 24 | 12.63 |
| 30 | 13.41 |
| 36 | 10.59 |
| 42 | 13.66 |After teriparatide discontinued
*p < 0.0001 compared to baseline
3
