# Supplementary material for: Effects of Teriparatide in Patients with Osteoporosis in Clinical Practice: 42-Month Results During and After Discontinuation of Treatment from the European Extended Forsteo® Observational Study (ExFOS)
Source: Calcif Tissue Int. 2018 Jun 16;103(4):359–71. doi: 10.1007/s00223-018-0437-x (PMC6153867; doi:10.1007/s00223-018-0437-x)
Supplement: Supplementary file 5 — Supplementary material 5 (PPTX 50 KB) [file 223_2018_437_MOESM5_ESM.pptx]

## Slide 1
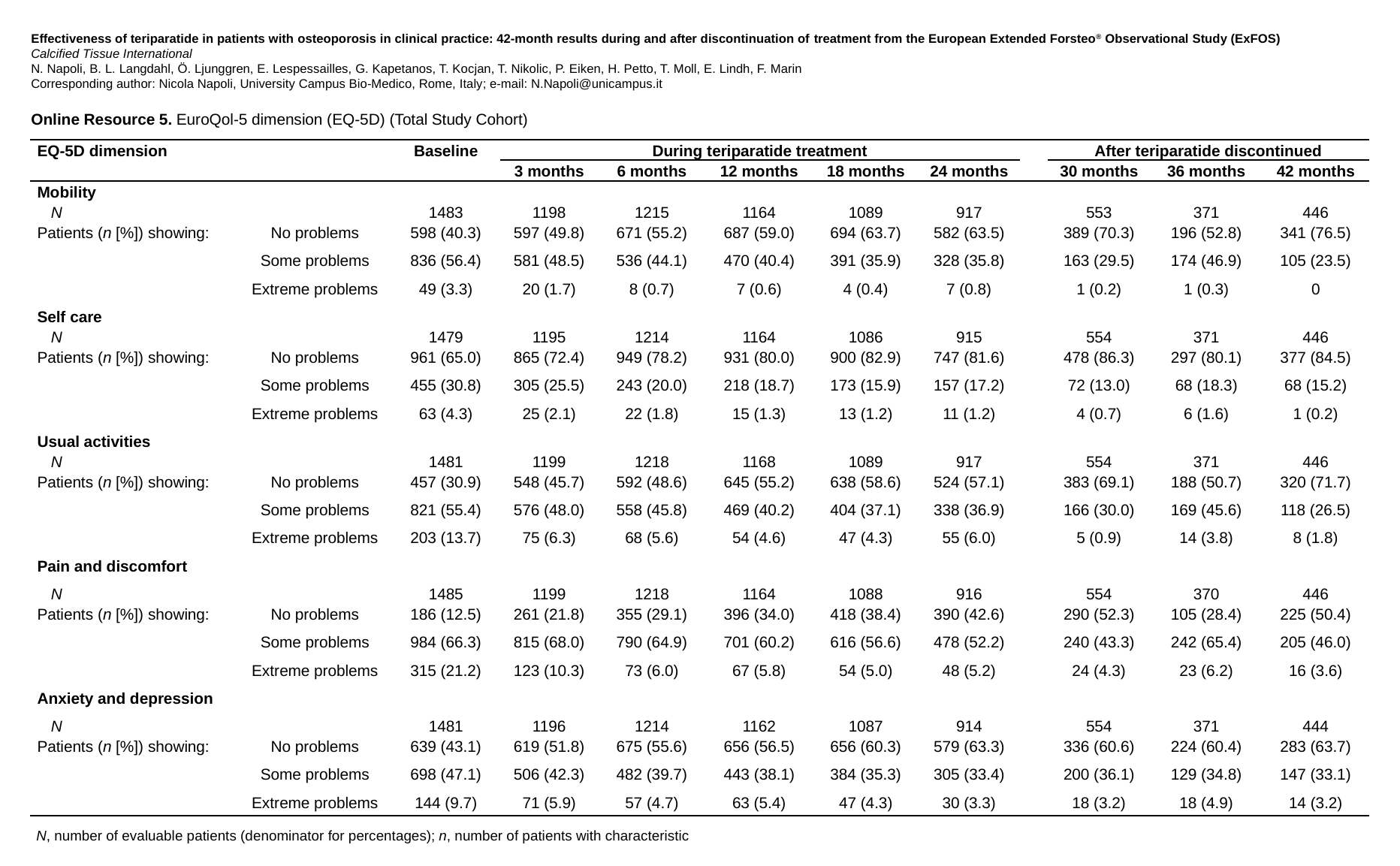

Effectiveness of teriparatide in patients with osteoporosis in clinical practice: 42-month results during and after discontinuation of treatment from the European Extended Forsteo® Observational Study (ExFOS)
Calcified Tissue International
N. Napoli, B. L. Langdahl, Ö. Ljunggren, E. Lespessailles, G. Kapetanos, T. Kocjan, T. Nikolic, P. Eiken, H. Petto, T. Moll, E. Lindh, F. Marin
Corresponding author: Nicola Napoli, University Campus Bio-Medico, Rome, Italy; e-mail: N.Napoli@unicampus.it
Online Resource 5. EuroQol-5 dimension (EQ-5D) (Total Study Cohort)
| EQ-5D dimension | | Baseline | During teriparatide treatment | | | | | | After teriparatide discontinued | | |
| --- | --- | --- | --- | --- | --- | --- | --- | --- | --- | --- | --- |
| | | | 3 months | 6 months | 12 months | 18 months | 24 months | | 30 months | 36 months | 42 months |
| Mobility | | | | | | | | | | | |
| N | | 1483 | 1198 | 1215 | 1164 | 1089 | 917 | | 553 | 371 | 446 |
| Patients (n [%]) showing: | No problems | 598 (40.3) | 597 (49.8) | 671 (55.2) | 687 (59.0) | 694 (63.7) | 582 (63.5) | | 389 (70.3) | 196 (52.8) | 341 (76.5) |
| | Some problems | 836 (56.4) | 581 (48.5) | 536 (44.1) | 470 (40.4) | 391 (35.9) | 328 (35.8) | | 163 (29.5) | 174 (46.9) | 105 (23.5) |
| | Extreme problems | 49 (3.3) | 20 (1.7) | 8 (0.7) | 7 (0.6) | 4 (0.4) | 7 (0.8) | | 1 (0.2) | 1 (0.3) | 0 |
| Self care | | | | | | | | | | | |
| N | | 1479 | 1195 | 1214 | 1164 | 1086 | 915 | | 554 | 371 | 446 |
| Patients (n [%]) showing: | No problems | 961 (65.0) | 865 (72.4) | 949 (78.2) | 931 (80.0) | 900 (82.9) | 747 (81.6) | | 478 (86.3) | 297 (80.1) | 377 (84.5) |
| | Some problems | 455 (30.8) | 305 (25.5) | 243 (20.0) | 218 (18.7) | 173 (15.9) | 157 (17.2) | | 72 (13.0) | 68 (18.3) | 68 (15.2) |
| | Extreme problems | 63 (4.3) | 25 (2.1) | 22 (1.8) | 15 (1.3) | 13 (1.2) | 11 (1.2) | | 4 (0.7) | 6 (1.6) | 1 (0.2) |
| Usual activities | | | | | | | | | | | |
| N | | 1481 | 1199 | 1218 | 1168 | 1089 | 917 | | 554 | 371 | 446 |
| Patients (n [%]) showing: | No problems | 457 (30.9) | 548 (45.7) | 592 (48.6) | 645 (55.2) | 638 (58.6) | 524 (57.1) | | 383 (69.1) | 188 (50.7) | 320 (71.7) |
| | Some problems | 821 (55.4) | 576 (48.0) | 558 (45.8) | 469 (40.2) | 404 (37.1) | 338 (36.9) | | 166 (30.0) | 169 (45.6) | 118 (26.5) |
| | Extreme problems | 203 (13.7) | 75 (6.3) | 68 (5.6) | 54 (4.6) | 47 (4.3) | 55 (6.0) | | 5 (0.9) | 14 (3.8) | 8 (1.8) |
| Pain and discomfort | | | | | | | | | | | |
| N | | 1485 | 1199 | 1218 | 1164 | 1088 | 916 | | 554 | 370 | 446 |
| Patients (n [%]) showing: | No problems | 186 (12.5) | 261 (21.8) | 355 (29.1) | 396 (34.0) | 418 (38.4) | 390 (42.6) | | 290 (52.3) | 105 (28.4) | 225 (50.4) |
| | Some problems | 984 (66.3) | 815 (68.0) | 790 (64.9) | 701 (60.2) | 616 (56.6) | 478 (52.2) | | 240 (43.3) | 242 (65.4) | 205 (46.0) |
| | Extreme problems | 315 (21.2) | 123 (10.3) | 73 (6.0) | 67 (5.8) | 54 (5.0) | 48 (5.2) | | 24 (4.3) | 23 (6.2) | 16 (3.6) |
| Anxiety and depression | | | | | | | | | | | |
| N | | 1481 | 1196 | 1214 | 1162 | 1087 | 914 | | 554 | 371 | 444 |
| Patients (n [%]) showing: | No problems | 639 (43.1) | 619 (51.8) | 675 (55.6) | 656 (56.5) | 656 (60.3) | 579 (63.3) | | 336 (60.6) | 224 (60.4) | 283 (63.7) |
| | Some problems | 698 (47.1) | 506 (42.3) | 482 (39.7) | 443 (38.1) | 384 (35.3) | 305 (33.4) | | 200 (36.1) | 129 (34.8) | 147 (33.1) |
| | Extreme problems | 144 (9.7) | 71 (5.9) | 57 (4.7) | 63 (5.4) | 47 (4.3) | 30 (3.3) | | 18 (3.2) | 18 (4.9) | 14 (3.2) |
N, number of evaluable patients (denominator for percentages); n, number of patients with characteristic
