# Supplementary material for: Effects of Teriparatide in Patients with Osteoporosis in Clinical Practice: 42-Month Results During and After Discontinuation of Treatment from the European Extended Forsteo® Observational Study (ExFOS)
Source: Calcif Tissue Int. 2018 Jun 16;103(4):359–71. doi: 10.1007/s00223-018-0437-x (PMC6153867; doi:10.1007/s00223-018-0437-x)
Supplement: Supplementary file 6 — Supplementary material 6 (PPTX 49 KB) [file 223_2018_437_MOESM6_ESM.pptx]

## Slide 1
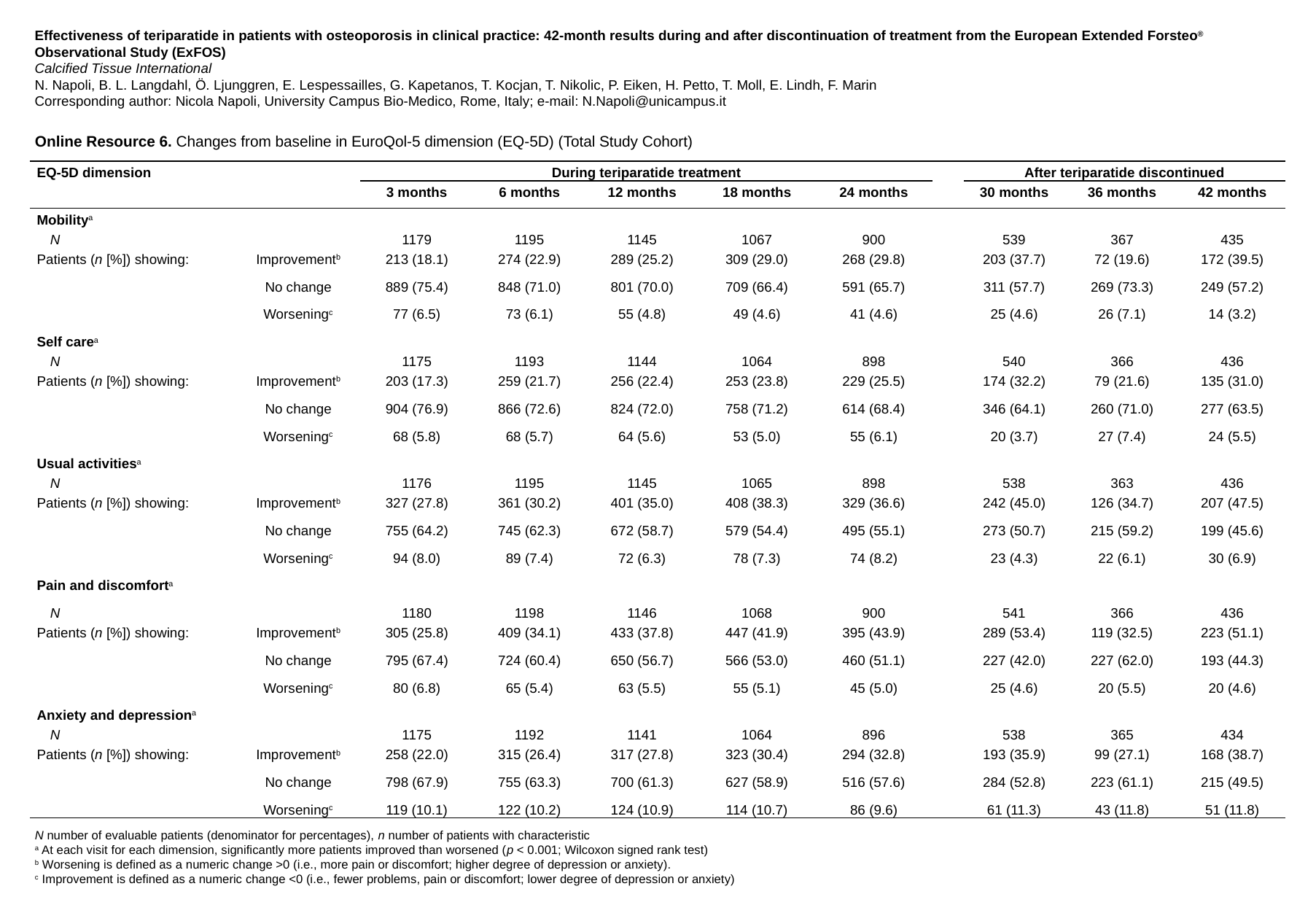

Effectiveness of teriparatide in patients with osteoporosis in clinical practice: 42-month results during and after discontinuation of treatment from the European Extended Forsteo® Observational Study (ExFOS)
Calcified Tissue International
N. Napoli, B. L. Langdahl, Ö. Ljunggren, E. Lespessailles, G. Kapetanos, T. Kocjan, T. Nikolic, P. Eiken, H. Petto, T. Moll, E. Lindh, F. Marin
Corresponding author: Nicola Napoli, University Campus Bio-Medico, Rome, Italy; e-mail: N.Napoli@unicampus.it
Online Resource 6. Changes from baseline in EuroQol-5 dimension (EQ-5D) (Total Study Cohort)
| EQ-5D dimension | | During teriparatide treatment | | | | | | After teriparatide discontinued | | |
| --- | --- | --- | --- | --- | --- | --- | --- | --- | --- | --- |
| | | 3 months | 6 months | 12 months | 18 months | 24 months | | 30 months | 36 months | 42 months |
| Mobilitya | | | | | | | | | | |
| N | | 1179 | 1195 | 1145 | 1067 | 900 | | 539 | 367 | 435 |
| Patients (n [%]) showing: | Improvementb | 213 (18.1) | 274 (22.9) | 289 (25.2) | 309 (29.0) | 268 (29.8) | | 203 (37.7) | 72 (19.6) | 172 (39.5) |
| | No change | 889 (75.4) | 848 (71.0) | 801 (70.0) | 709 (66.4) | 591 (65.7) | | 311 (57.7) | 269 (73.3) | 249 (57.2) |
| | Worseningc | 77 (6.5) | 73 (6.1) | 55 (4.8) | 49 (4.6) | 41 (4.6) | | 25 (4.6) | 26 (7.1) | 14 (3.2) |
| Self carea | | | | | | | | | | |
| N | | 1175 | 1193 | 1144 | 1064 | 898 | | 540 | 366 | 436 |
| Patients (n [%]) showing: | Improvementb | 203 (17.3) | 259 (21.7) | 256 (22.4) | 253 (23.8) | 229 (25.5) | | 174 (32.2) | 79 (21.6) | 135 (31.0) |
| | No change | 904 (76.9) | 866 (72.6) | 824 (72.0) | 758 (71.2) | 614 (68.4) | | 346 (64.1) | 260 (71.0) | 277 (63.5) |
| | Worseningc | 68 (5.8) | 68 (5.7) | 64 (5.6) | 53 (5.0) | 55 (6.1) | | 20 (3.7) | 27 (7.4) | 24 (5.5) |
| Usual activitiesa | | | | | | | | | | |
| N | | 1176 | 1195 | 1145 | 1065 | 898 | | 538 | 363 | 436 |
| Patients (n [%]) showing: | Improvementb | 327 (27.8) | 361 (30.2) | 401 (35.0) | 408 (38.3) | 329 (36.6) | | 242 (45.0) | 126 (34.7) | 207 (47.5) |
| | No change | 755 (64.2) | 745 (62.3) | 672 (58.7) | 579 (54.4) | 495 (55.1) | | 273 (50.7) | 215 (59.2) | 199 (45.6) |
| | Worseningc | 94 (8.0) | 89 (7.4) | 72 (6.3) | 78 (7.3) | 74 (8.2) | | 23 (4.3) | 22 (6.1) | 30 (6.9) |
| Pain and discomforta | | | | | | | | | | |
| N | | 1180 | 1198 | 1146 | 1068 | 900 | | 541 | 366 | 436 |
| Patients (n [%]) showing: | Improvementb | 305 (25.8) | 409 (34.1) | 433 (37.8) | 447 (41.9) | 395 (43.9) | | 289 (53.4) | 119 (32.5) | 223 (51.1) |
| | No change | 795 (67.4) | 724 (60.4) | 650 (56.7) | 566 (53.0) | 460 (51.1) | | 227 (42.0) | 227 (62.0) | 193 (44.3) |
| | Worseningc | 80 (6.8) | 65 (5.4) | 63 (5.5) | 55 (5.1) | 45 (5.0) | | 25 (4.6) | 20 (5.5) | 20 (4.6) |
| Anxiety and depressiona | | | | | | | | | | |
| N | | 1175 | 1192 | 1141 | 1064 | 896 | | 538 | 365 | 434 |
| Patients (n [%]) showing: | Improvementb | 258 (22.0) | 315 (26.4) | 317 (27.8) | 323 (30.4) | 294 (32.8) | | 193 (35.9) | 99 (27.1) | 168 (38.7) |
| | No change | 798 (67.9) | 755 (63.3) | 700 (61.3) | 627 (58.9) | 516 (57.6) | | 284 (52.8) | 223 (61.1) | 215 (49.5) |
| | Worseningc | 119 (10.1) | 122 (10.2) | 124 (10.9) | 114 (10.7) | 86 (9.6) | | 61 (11.3) | 43 (11.8) | 51 (11.8) |
N number of evaluable patients (denominator for percentages), n number of patients with characteristic
a At each visit for each dimension, significantly more patients improved than worsened (p < 0.001; Wilcoxon signed rank test)
b Worsening is defined as a numeric change >0 (i.e., more pain or discomfort; higher degree of depression or anxiety).
c Improvement is defined as a numeric change <0 (i.e., fewer problems, pain or discomfort; lower degree of depression or anxiety)
