# Supplementary material for: Effects of Teriparatide in Patients with Osteoporosis in Clinical Practice: 42-Month Results During and After Discontinuation of Treatment from the European Extended Forsteo® Observational Study (ExFOS)
Source: Calcif Tissue Int. 2018 Jun 16;103(4):359–71. doi: 10.1007/s00223-018-0437-x (PMC6153867; doi:10.1007/s00223-018-0437-x)
Supplement: Supplementary file 7 — Supplementary material 7 (PPTX 43 KB) [file 223_2018_437_MOESM7_ESM.pptx]

## Slide 1
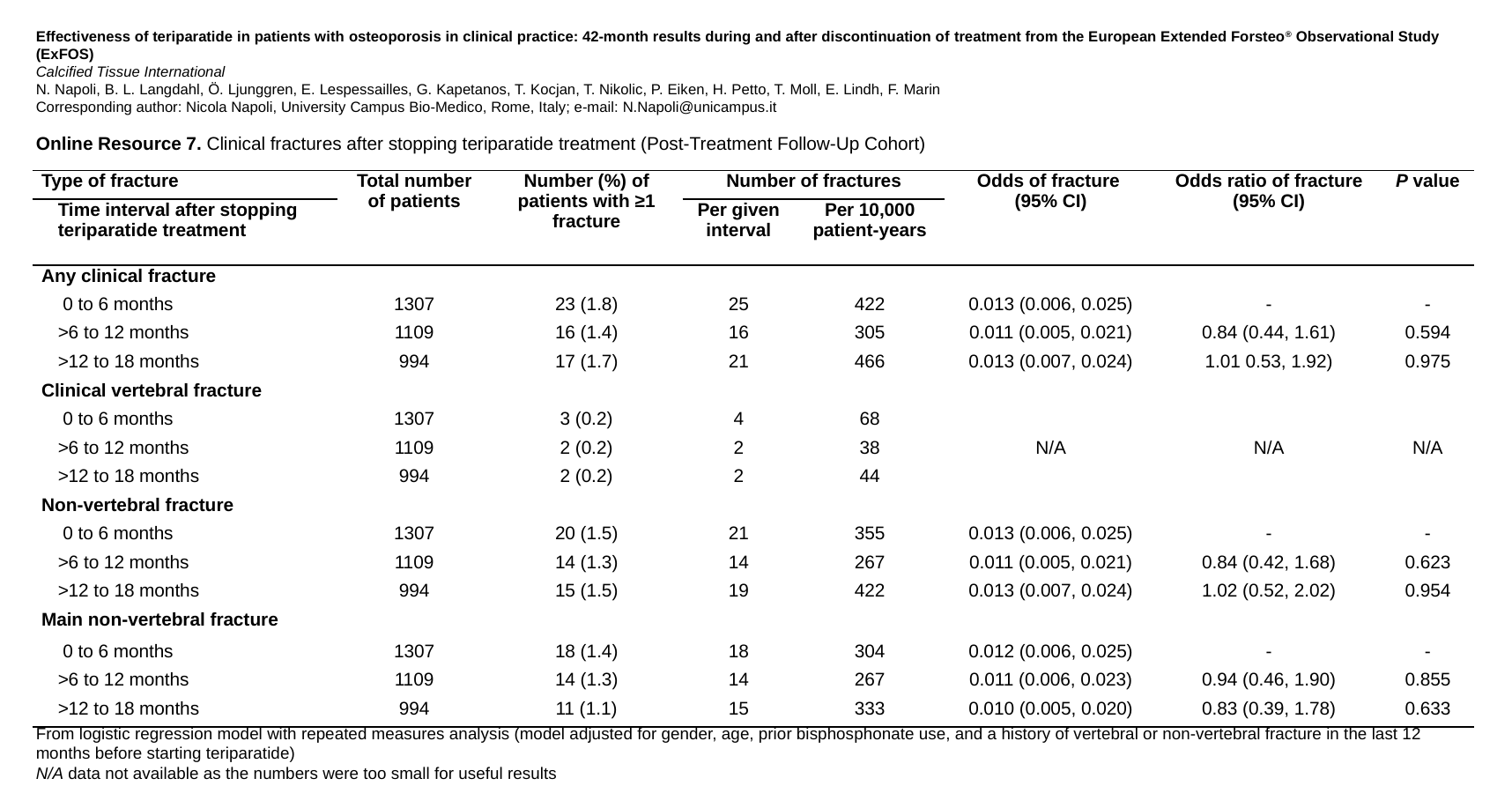

Effectiveness of teriparatide in patients with osteoporosis in clinical practice: 42-month results during and after discontinuation of treatment from the European Extended Forsteo® Observational Study (ExFOS)
Calcified Tissue International
N. Napoli, B. L. Langdahl, Ö. Ljunggren, E. Lespessailles, G. Kapetanos, T. Kocjan, T. Nikolic, P. Eiken, H. Petto, T. Moll, E. Lindh, F. Marin
Corresponding author: Nicola Napoli, University Campus Bio-Medico, Rome, Italy; e-mail: N.Napoli@unicampus.it
Online Resource 7. Clinical fractures after stopping teriparatide treatment (Post-Treatment Follow-Up Cohort)
| Type of fracture | Total number of patients | Number (%) of patients with ≥1 fracture | Number of fractures | | Odds of fracture (95% CI) | Odds ratio of fracture (95% CI) | P value |
| --- | --- | --- | --- | --- | --- | --- | --- |
| Time interval after stopping teriparatide treatment | | | Per given interval | Per 10,000 patient-years | | | |
| Any clinical fracture | | | | | | | |
| 0 to 6 months | 1307 | 23 (1.8) | 25 | 422 | 0.013 (0.006, 0.025) | - | - |
| >6 to 12 months | 1109 | 16 (1.4) | 16 | 305 | 0.011 (0.005, 0.021) | 0.84 (0.44, 1.61) | 0.594 |
| >12 to 18 months | 994 | 17 (1.7) | 21 | 466 | 0.013 (0.007, 0.024) | 1.01 0.53, 1.92) | 0.975 |
| Clinical vertebral fracture | | | | | | | |
| 0 to 6 months | 1307 | 3 (0.2) | 4 | 68 | | | |
| >6 to 12 months | 1109 | 2 (0.2) | 2 | 38 | N/A | N/A | N/A |
| >12 to 18 months | 994 | 2 (0.2) | 2 | 44 | | | |
| Non-vertebral fracture | | | | | | | |
| 0 to 6 months | 1307 | 20 (1.5) | 21 | 355 | 0.013 (0.006, 0.025) | - | - |
| >6 to 12 months | 1109 | 14 (1.3) | 14 | 267 | 0.011 (0.005, 0.021) | 0.84 (0.42, 1.68) | 0.623 |
| >12 to 18 months | 994 | 15 (1.5) | 19 | 422 | 0.013 (0.007, 0.024) | 1.02 (0.52, 2.02) | 0.954 |
| Main non-vertebral fracture | | | | | | | |
| 0 to 6 months | 1307 | 18 (1.4) | 18 | 304 | 0.012 (0.006, 0.025) | - | - |
| >6 to 12 months | 1109 | 14 (1.3) | 14 | 267 | 0.011 (0.006, 0.023) | 0.94 (0.46, 1.90) | 0.855 |
| >12 to 18 months | 994 | 11 (1.1) | 15 | 333 | 0.010 (0.005, 0.020) | 0.83 (0.39, 1.78) | 0.633 |
From logistic regression model with repeated measures analysis (model adjusted for gender, age, prior bisphosphonate use, and a history of vertebral or non-vertebral fracture in the last 12 months before starting teriparatide)
N/A data not available as the numbers were too small for useful results
